# A comparative analysis of host responses to avian influenza infection in ducks and chickens highlights a role for the interferon-induced transmembrane proteins in viral resistance

**DOI:** 10.1186/s12864-015-1778-8

**Published:** 2015-08-04

**Authors:** Jacqueline Smith, Nikki Smith, Le Yu, Ian R. Paton, Maria Weronika Gutowska, Heather L. Forrest, Angela F. Danner, J. Patrick Seiler, Paul Digard, Robert G. Webster, David W. Burt

**Affiliations:** The Roslin Institute and R(D)SVS, University of Edinburgh, Easter Bush, Midlothian, EH25 9RG UK; St. Jude Children’s Research Hospital, Virology Division, Department of Infectious Diseases, 262 Danny Thomas Place, Memphis, TN 38105 USA

**Keywords:** Avian influenza, H5N1, H5N2, Duck, Chicken, Pathogenicity, Interferon-induced transmembrane proteins, Evolution and gene expression profiles

## Abstract

**Background:**

Chickens are susceptible to infection with a limited number of Influenza A viruses and are a potential source of a human influenza pandemic. In particular, H5 and H7 haemagglutinin subtypes can evolve from low to highly pathogenic strains in gallinaceous poultry. Ducks on the other hand are a natural reservoir for these viruses and are able to withstand most avian influenza strains.

**Results:**

Transcriptomic sequencing of lung and ileum tissue samples from birds infected with high (H5N1) and low (H5N2) pathogenic influenza viruses has allowed us to compare the early host response to these infections in both these species. Chickens (but not ducks) lack the intracellular receptor for viral ssRNA, *RIG-I* and the gene for an important RIG-I binding protein, RNF135. These differences in gene content partly explain the differences in host responses to low pathogenic and highly pathogenic avian influenza virus in chicken and ducks. We reveal very different patterns of expression of members of the interferon-induced transmembrane protein (*IFITM*) gene family in ducks and chickens. In ducks, *IFITM1, 2* and *3* are strongly up regulated in response to highly pathogenic avian influenza, where little response is seen in chickens. Clustering of gene expression profiles suggests IFITM1 and 2 have an anti-viral response and IFITM3 may restrict avian influenza virus through cell membrane fusion. We also show, through molecular phylogenetic analyses, that avian *IFITM1* and *IFITM3* genes have been subject to both episodic and pervasive positive selection at specific codons. In particular, avian *IFITM1* showed evidence of positive selection in the duck lineage at sites known to restrict influenza virus infection.

**Conclusions:**

Taken together these results support a model where the IFITM123 protein family and RIG-I all play a crucial role in the tolerance of ducks to highly pathogenic and low pathogenic strains of avian influenza viruses when compared to the chicken.

**Electronic supplementary material:**

The online version of this article (doi:10.1186/s12864-015-1778-8) contains supplementary material, which is available to authorized users.

## Background

The question of how ducks can survive challenge by all low pathogenic avian influenza (LPAI) and most highly pathogenic avian influenza (HPAI) infections, yet chickens can only survive LPAI, remains an important question due to the economic losses experienced by the poultry industry, the human health implications and the continuing threat of pandemic disease posed by avian viruses. Most avian influenza strains are able to infect and replicate in ducks, and are usually asymptomatic and seldom cause disease. Of the 16 haemagglutinin subtypes of influenza viruses infecting migratory waterfowl, the H5 and H7 subtypes are unique. After transmission to gallinaceous poultry the H5 and H7 viruses can evolve into highly pathogenic strains. In ducks and other aquatic birds the low pathogenic H5 and H7 influenza viruses replicate predominantly in the intestinal tract without any outward clinical signs of infection. After transmission to gallinaceous poultry and acquisition of multiple basic amino acids in the connecting peptide of the haemagglutinin, the viruses become highly pathogenic and replicate systemically in the chicken [1]. HPAI has proven to be deadly to chickens within a very short time frame post-infection, although LPAI only produces mild or no signs of clinical disease [2]. Ducks on the other hand are able to mount a robust inflammatory response against most HPAI [3], although they have shown increased susceptibility to some emerging strains of H5N1, with death resulting in some cases [4–6]. The difference in pathogenesis seen between ducks and chickens could also be due to the fact that a rapid induction of apoptosis in HPAI-infected ducks may be beneficial to the host, whereas delayed apoptosis in chickens may be an advantage for the virus [7].

HPAI H5N1 remains a concern, with new strains continually evolving and increasing the pandemic threat from this subtype. Worryingly, the recently emerged H7N9 subtype also poses a risk of being the vector by which a human influenza epidemic occurs. The first human infections by this virus were reported early in 2013, and by June 2013, the mortality rate was over 30 % - a level still being seen as cases continue to be reported. The World Health Organization (WHO) has identified H7N9 as "…an unusually dangerous virus for humans” [8]. Although not posing an imminent threat, H10N8 has also recently been identified as having the potential to spread from birds to humans if the necessary viral mutations occur [9].

Viral haemagglutinin binds to cell surface receptors in susceptible host cells. In humans, virus binds to sialic acid α2, 6-galactose (SAα2, 6-Gal) linked receptors whereas avian viruses preferentially bind to sialic acid α2, 3-galactose (SAα2, 3-Gal) linked receptors [10, 11]. SAα2, 6-Gal linked receptors are the predominant type in the trachea of chickens, while duck tracheas contain more SAα2, 3-Gal linked receptors [12]. This may be one reason why chickens have the potential to act as an intermediate host for human infection.

The avian response to infection compared to that of humans is also determined by their immune gene repertoire. It is known that birds have a much more compact set of immune-related genes than mammals [13, 14]. With the increasing availability of avian genome sequences, it is also becoming apparent that key genes are reportedly missing in some birds, which will affect their host responses to viral infections. These include Toll-like receptors 8 and 9 (*TLR8* and *TLR9*) that, in humans, recognize ssRNA and CpG, respectively [15, 16]. Interferon-stimulated gene 15 (*ISG15*), interferon regulatory factor 3 (*IRF3*) and *TNFα* are all genes which have not yet been identified in birds [17]. When we compare the genomes of chickens and ducks we can also see differences in their immune gene complement. Crucially, chickens (but not ducks) appear to be lacking the intracellular receptor for viral ssRNA, *RIG-I* (also called *DDX58*) [18] and the gene for an important RIG-I binding protein, RNF135 [17]. These differences may also partly explain the differences seen between chickens and ducks in their responses towards avian influenza infections [17].

One family of genes which are known to have a role in limiting influenza infection in mammals is the interferon induced transmembrane protein (IFITM) family [19, 20]. These genes are part of a larger family called the Dispanins which have a common double transmembrane domain configuration [21]. In humans, *IFITM1*, *2*, *3*, *5* and *10* have been identified. The gene cluster *IFITM1, 2* and *3* are known to have anti-viral function (the “immune-related” or IR-IFITM gene cluster; [22]), IFITM5 may be involved in bone mineralization and the role of IFITM10 is less clear [23]. Although exactly how they function has not been fully elucidated, IFITM proteins have been found to be enriched in late endosomes and lysosomes and are thought to act before viral membrane fusion occurs [24]. Human IFITM3 has been shown to block viral entry into the host cell [25], with a requirement of the CD225 domain for inhibition of the influenza virus [26]. Until now, most of these genes remained unannotated in the chicken and duck genomes and their role in avian influenza infections was unknown.

To examine the role, if any, of the *IFITM* gene family we searched for members in the genomes of the chicken, duck and other avian species, performed an in depth molecular phylogenetic analyses to detect positive selection acting on specific codons and then compared the expression of host genes following infection by low and high pathogenic strains of avian influenza viruses in ducks and chickens. In this study we examine and compare the expression of innate immune related genes in chickens and ducks after infection with both LPAI (H5N2) and HPAI (H5N1); these being avian species showing high (usually leading to death) and low susceptibility (mostly asymptomatic), respectively. We looked at the host immune response and viral replication in the ileum and lung 1 and 3 days post infection (dpi). HPAI viruses replicate primarily in the respiratory tract prior to systemic spread with generalized replication and death, while low pathogenic viruses replicate primarily in the intestinal tract and to a limited extent in the respiratory tract with no apparent disease signs [27]. Dramatic differences in host responses to avian influenza infection were found in chickens and ducks. In ducks *IFITM1, 2* and *3* are strongly up regulated in response to HPAI virus, where little response is seen in chickens. Clustering of gene expression profiles suggests IFITM1 and 2 have an anti-viral response and IFITM3 may act before viral membrane fusion occurs and thus blocks viral entry. We also show through molecular phylogenetic analyses that avian *IFITM1* and *3* genes have been subject to both episodic and pervasive positive selection at specific codons. Specifically, avian *IFITM1* shows evidence of positive selection in the duck lineage at a site known to restrict avian influenza virus (AIV) infection. Overexpression of this gene *in vitro* has been shown to increase the resistance of avian cells to AIV infection [28], probably by a block in membrane fusion, crucial to the entry and further replication of this virus. Taken together these results support a model where the IFITM123 family and RIG-I play a crucial role in the tolerance of ducks to high and low pathogenic strains of avian influenza viruses when compared to the chicken and other Galliformes.

## Results

### Characterisation of the *IFITM* genes in the genomes of chicken and duck

The *IFITM* response to influenza infection has not been examined *in vivo* in any avian species. We therefore resolved to identify and annotate these genes in the chicken and duck genomes, examine their evolution in vertebrates and determine their expression after infection with either LPAI or HPAI viruses. This would allow the evolution of the *IFITM* gene family and their response to influenza infection to be compared with mammals and between chickens and ducks. This analysis would highlight similarities and differences that may be correlated with susceptibility to infection by this group of viruses.

With only the *IFITM3-like*, *IFITM5* and part of the *IFITM10* genes annotated in the chicken genome we had to first identify the genomic locations of other chicken *IFITM* genes (if any) and identify the orthologous genes on the relevant scaffolds in the sequenced duck genome. The locations of the genes in the chicken genome (Galgal4; GCA_000002315.2) were determined to be clustered on Chr5 as follows: c*IFITM1*: 1592913–1593904; c*IFITM2*: 1598390–1599631; c*IFITM3*: 1596326–1597707; c*IFITM5*: 1600790–1601575 and c*IFITM10*: 13520526–13526834. The chicken *IFITM* locus was previously described by Smith et al. [28] although the true orthology with mammals was uncertain. With respect to the avian gene nomenclature, *IFITM1* has been annotated as ‘*chIFITM3*’ by these authors and *IFITM3* was called ‘*chIFITM1*’.

Aside from *IFITM5*, only partial duck *IFITM* gene sequences could be identified in the duck genome (BGI_duck_1.0; GCA_000355885.1) using the chicken sequences as probes in any Blast homology searches. The complete *IFITM1* gene was identified in the duck genome using the chicken sequence based on a Genewise prediction and the full coding sequence was confirmed by 5’-RACE. The complete cDNA sequences for duck *IFITM2, 3* and *10* required cloning by 5’-RACE experiments to complete the 5’-end of each sequence. The *IFITM5* gene was also confirmed by sequencing of the genomic locus. Analysis of the protein sequences using the SMART algorithm showed that both the chicken and duck proteins share the same double transmembrane domain structure and the highly conserved CD225 domain, as found in other species [22] (Additional file 1: Figure S1). Comparison of duck and chicken IFITM protein sequences shows varying degrees of sequence identity: IFITM1 40 %, IFITM2 68 %, IFITM3 75 %, IFITM5 92 % and IFITM10 92 %. (All duck sequences have been submitted to the public databases under the following accession numbers: IFITM1: GenBank- KF584226; IFITM2: GenBank- KF584227; IFITM3: GenBank- KF584228; IFITM5: EMBL- HG764554; IFITM10: GenBank- KF584229).

### Evolutionary relationships of *IFITM* genes in birds, mammals and amphibians

In mammals the highly conserved IFITM5 and 10 proteins cluster into distinct evolutionary groups, while a large number of lineage and species-specific gene duplications were observed in the IR-IFITM sub-family; made up of IFITM1, 2 and 3-like proteins [22]. For example, these authors were able to define expansions specific to rodent and primate clades. Gene conversion was previously reported in a few species including dog, cow and horse but is not a factor in other species, including birds [22].

In order to study the evolution of the avian *IFITM* gene family and elucidate gene orthologs with other vertebrates, *IFITM* coding sequences were collected from avian species based on previously annotated genes or homology searches of the 48 avian genomes analysed by the *Avian Phylogenetics Consortium* using the chicken and duck IFITM sequences as probes (see METHODS, Additional file 2: Table S1 describes the source of the sequences and Additional file 3: Table S2 lists the species names and abbreviations). In addition, IFITM sequences from mammals, non-avian reptiles and amphibians were downloaded from GenBank (NCBI) and used to compare with avian homologs.

**Fig. 1:**
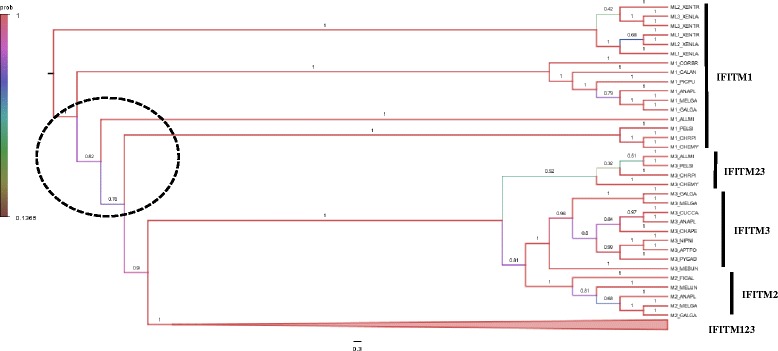
Bayesian tree of the vertebrate *IFITM1, 2* and *3*-like gene family. A Bayesian tree was constructed using MrBayes (v3.2.2) with 1 million generations. Branch confidence values are shown at the nodes and coloured using the probability scale on the left. Scale bar corresponds to 0.3 substitutions per site. IFITM123 represent the mammalian IFITM1, 2 and 3-like genes and for simplicity are shown as a triangle. Details of the mammalian tree are shown in Additional file 7: Figure S5D. All the other IFITM genes are derived from avian, non-avian reptiles and amphibians. The species abbreviations are shown on Additional file 3: Table S2. The dotted circle indicates uncertainty in the split near the root of the tree

Using conservation of gene order we were able to define 1:1 orthologs between the avian and mammalian *IR-IFITM* gene cluster, and the *IFITM5* and *10* genes. These three groups of IFITM sequences were used to create a multiple sequence alignment (Additional file 4: Figure S2) and in the construction of a phylogenetic gene tree (Additional file 5: Figure S3). As expected, the *IFITM5* and *10* genes cluster into distinct clades in birds, other non-avian reptiles, mammals and amphibians but the picture for the *IR-IFITM* gene family was more complex. In birds, the three *IR-IFITM* genes cluster into three distinct groups of *IFITM1*, *2* and *3* sequences. In mammals, however the genes tend to cluster together within their own species or clades, indicating more recent clade-specific gene expansions, as shown before by Zhang et al. [22] making it impossible to define 1:1 orthologs between avian and mammalian *IFITM1*, 2 and 3-like genes. In an attempt to resolve this issue and define the gene duplication events that gave rise to this family, we prepared a multiple sequence alignment of the IR-IFITM protein family. To increase the power of our analysis we collected IFITM1, 2 and 3-like peptide sequences from a wide range of vertebrates including birds, non-avian reptiles, mammals and amphibians. After multiple sequence alignment (MSA) using MUSCLE, we removed partial, duplicated or highly divergent (likely to be sequencing errors or incorrect spliced products) peptide sequences, after which 148 peptide sequences remained in the final multiple sequence alignment (Additional file 6: Figure S4). MrBayes, a Bayesian package for inference [29, 30], was used to infer phylogenetic trees from the MSA of IFITM1/2/3-like peptide sequences (Fig. 1). There appeared to be a single clade of *IFITM1*-like genes in the amphibians (Xenopus species), which suggested an independent origin from birds and mammals. The Xenopus sequences were therefore used to root the gene tree. The tree showed strong support for independent *IFITM* gene duplications in mammals and reptiles. In mammals, as Zhang et al. [22] have shown previously, there were a number of clade-specific expansions of this gene family. However in birds, there were only two gene duplications that gave rise to the avian *IFITM1*, *2* and *3* genes. The *IFITM2* and *3* genes appear to have arisen from an avian-specific gene duplication, with only a single *IFITM2/3*-like gene in non-avian reptiles. The *IFITM1* gene appears to have arisen from an earlier gene duplication in all reptiles. This Bayesian analysis was supported by similar analyses based on Maximum Likelihood, Neighbour-Joining and Parsimony methods, as implemented in the MEGA6 package (Additional file 7: Figure S5) using the JTT + G_I model predicted as the most likely substitution model (Additional file 8: Table S3). These results also show that the structure and function of the avian IFITM123 protein family is most likely to share elements of all the human IFITM1, 2 and 3 proteins of the *IR-IFITM* gene cluster.

### Positive selection acting on the *IFITM* subfamilies

In mammals, it is known that the *IFITM5* and *10* genes are highly conserved and the genes of the *IR-IFITM* gene cluster are more divergent and predicted to be under positive selection [22]. We used methods implemented in DATAMONKEY [31] and CODEML to investigate whether positive selection has also driven the evolution of the avian *IFITM* gene family. Gene conversion was found in some mammals under species-specific duplication, including dog, cow, horse, etc. [22]. These sequences were removed from all subsequent analyses. No gene conversion was found in the avian *IFITM* gene family members [22].

MEME was used to seek evidence of positive selection at sites in the coding sequences of the IFITM gene family (Table 1). MEME is a method implemented within the DATAMONKEY package [32] that can identify both episodic and persistent positive selection because it allows the distribution of the dN/dS ratio to vary from site to site and from branch to branch at a site. In birds, only two genes - *IFITM1* and *3* show sites with strong evidence of positive selection. For *IFITM1*, most sites (6 codons) were found in the second transmembrane domain (TM2). In addition, one site was found in each of the N- (codon 30) and C- (codon 135) termini of the IFITM1 peptide. Only two sites (codons 106 and 112) were predicted to show persistent positive selection in all avian branches. Most sites showed evidence of episodic positive selection in one (codon 30 ANAPL at a site known to restrict influenza viruses [26], codon 100 PHALE and codon 117 CALAN) or a few species (codons 102, 118 and 135). These results were supported by FUBAR [33], which detected persistent sites of positive selection at codons 106, 112, 118 and 135, also detected by MEME (Additional file 9: Table S4). No significant sites were found using CODEML for *IFITM1* (Additional file 10: Table S5), suggesting most were episodic sites. The other gene, *IFITM3,* was predicted to have fewer positively selected sites (only 3 codons), in one (codon 98 APTFO and codon 136 CUCCA) or more species (codon 119). Again, the transmembrane TM2 domain contained most sites (2/3 of cases). CODEML predicted two additional sites of persistent positive selection in both *IFITM3* and *10* (2 in the N-terminus) (Additional file 10: Table S5). These results suggest that the TM2 domain is likely to be important in recognition of pathogen-associated lipids. Of great interest was positive selection at codon 30 (Thr - > Pro) in ducks, at a site known to restrict influenza viruses [26].

**Table 1:** Positively selected sites in IR-IFITM genes detected using MEME method implemented in DATAMONKEY

Gene	Codon (AA)	Region	α	β^−^	Pr[β = β^−^]	β^+^	Pr[β = β^+^]	*p*-value	Posterior Probability w+ > 1
IFITM1	30 V	N-term	0.00	0.00	9.38E-01	71.79	0.60	0.002	ANAPL**
	100 L	IM2	0.00	0.00	8.35E-01	6.49	0.17	0.015	PHALE**
	102 L	IM2	0.36	0.00	7.62E-01	9.23	0.24	0.050	EGRGA**, LEPDI**, CARCR*
	106 F	IM2	0.00	0.00	1.00E-09	1.30	1.00	0.035	ALL**
	112A	IM2	0.00	0.00	1.00E-09	1.43	1.00	0.020	ALL**
	117G	IM2	0.00	0.00	9.00E-01	173.17	0.10	0.032	CALAN**
	118 T	IM2	0.00	0.00	3.73E-01	2.06	0.63	0.024	MESUN**, PHALE**, CALUM**, CUCCU**
	135P	C-term	0.00	0.00	3.63E-01	5.84	0.64	0.007	CHAPE**, PELCR**, EGRGA**, HALAL**, COLST**
IFITM3	98A	CLIP	0.63	0.00	9.28E-01	94.87	0.07	0.048	APTFO*
	119 L	IM2	0.00	0.00	3.82E-01	4.31	0.62	0.050	ANAPL**, MESUN, NIPNI**, PYGAD**, GALGA
	136I	IM2	0.00	0.00	8.35E-01	14.92	0.16	0.004	CUCCA**

This summary table reports the distribution of synonymous (α) and non-synonymous (β) substitution rates over sites inferred by the MEME model, where the proportion of branches with β > α is significantly greater than 0; p-value is derived using a mixture of *χ*2 distributions, **p-value < 0.01; *p-value < 0.05

### Transcriptomic analysis of host-responses to LPAI and HPAI viruses

With a view to analysing the *in vivo* expression of the *IFITM* genes after influenza infection in chicken and in duck, transcriptomic sequencing experiments were undertaken. This not only allowed us to determine the profile of *IFITM* gene expression, but also defined the host innate immune response after influenza infection in each species and allowed a comparison between species to be made. Since LPAI viruses from ducks are the ultimate source of HPAI viruses in chickens we used a LPAI H5N2 and a HPAI H5N1 strain to examine the host response to each class of influenza virus. This study involved the use of two different tissues known to be targets of viral infection (lung and ileum) in three different conditions (uninfected control or infected with either LPAI H5N2 or HPAI H5N1) at two different time points (1 dpi and 3 dpi), which would allow us to study the host response in each species. Differential expression of genes was thus determined for 12 different experimental scenarios in each of duck and chicken, as outlined in Table 2, with the main interest being on the differences that would be seen between duck and chicken. The numbers of genes regarded as significantly differentially expressed, FDR <0.05 and which have a fold-change >1.5 are listed. Additional file 11: Table S6 presents the significantly differentially expressed (DE) genes in the 12 comparisons for each of chicken and duck tissues.

**Table 2:** Number of differentially expressed genes in duck and chicken in each condition tested

			Chicken	Duck
Tissue	Day (pi)	Comparison	DE* genes (FC > 1.5)	DE* genes (FC > 1.5)
Ileum	day 1	Control vs. HPAI	643	321
		Control vs. LPAI	12	49
		LPAI vs. HPAI	194	105
	day 3	Control vs. HPAI	10	1101
		Control vs. LPAI	3	102
		LPAI vs. HPAI	13	270
Lung	day 1	Control vs. HPAI	43	1774
		Control vs. LPAI	40	69
		LPAI vs. HPAI	84	1984
	day 3	Control vs. HPAI	11	1118
		Control vs. LPAI	554	32
		LPAI vs. HPAI	702	900

FC, fold change; *FDR < 0.05

### Virology and pathogenesis of host-responses to LPAI and HPIA viruses

The LPAI H5N2 virus (A/Mallard/British Columbia/500/2005) was not adapted to chickens and required 10^6^ egg infectious doses of virus to infect 50 % (EID_50_) of birds. Nevertheless in the infected chickens the virus replicated to moderate titres in the lungs (10 ^1.75^-10 ^3.25^ EID_50_) and higher titres in the ileum (10 ^3.5^-10 ^5.5^ EID_50_) and caused no disease signs. In ducks the LPAI H5N2 replicated to low titres in the lungs (10 ^0.75^EID_50_) and higher titres in the ileum (10 ^3.0^-10 ^6.75^EID_50_) with no disease signs. In contrast, as little as 10^1.5^EID_50_ of the HPAI H5N1 (A/Vietnam/1203/04) killed all inoculated chickens between 2 and 5 dpi. Despite 100 % mortality we detected virus in only 1/5 birds 3 dpi. Ducks were infected with 10^6^EID_50_ of HPAI H5N1; the virus replicated to high titres in the lungs and ileum and 6/10 ducks died between 4 and 6 dpi. Viral doses were chosen to reflect earlier studies [2, 18].

### The chicken host response to avian influenza infection

Knowing that chickens react very differently to LPAI (H5N2) and HPAI (H5N1) viruses, it was expected that very different gene expression profiles would be seen after each infection. From the numbers of DE genes (Table 2), it can be seen that the chicken mounts a large response to H5N1 in the ileum by 1 dpi . However, this is short-lived and is gone by 3 dpi. This response against H5N1 is not reflected in the lung. The highly pathogenic H5N1 appears to prove too much for the chicken - the response seen early in the ileum is not sustained, and the birds succumb to the disease. A strong response is seen in the lung by 3 dpi, after H5N2 infection. The ileum is not particularly affected by H5N2 infection and the robust response seen in the lung is obviously able to counter the effects of the virus, stop further spread, and the chicken is able to survive the LPAI infection.

In order to examine which biological pathways are involved during the chicken response, the DE genes in ileum at 1 dpi during H5N1 infection and in the lung at 3 dpi during H5N2 infection were analysed. Additional file 12: Tables S7A and 7B show pathways which are significantly enriched in DE genes from ileum and lung, respectively. Along with processes such as antigen processing and phosphatidylinositol signalling, it can be seen that genes involved in axon guidance are perturbed during H5N1 infection in the ileum and B- and T-cell receptor signalling is activated in the lung against H5N2. Additional file 13: Figure S6 shows the genes being activated/inhibited in these pathways. The highlighted genes are also involved in natural killer cell activation, which is a process which will be occurring during the early response.

**Fig. 2:**
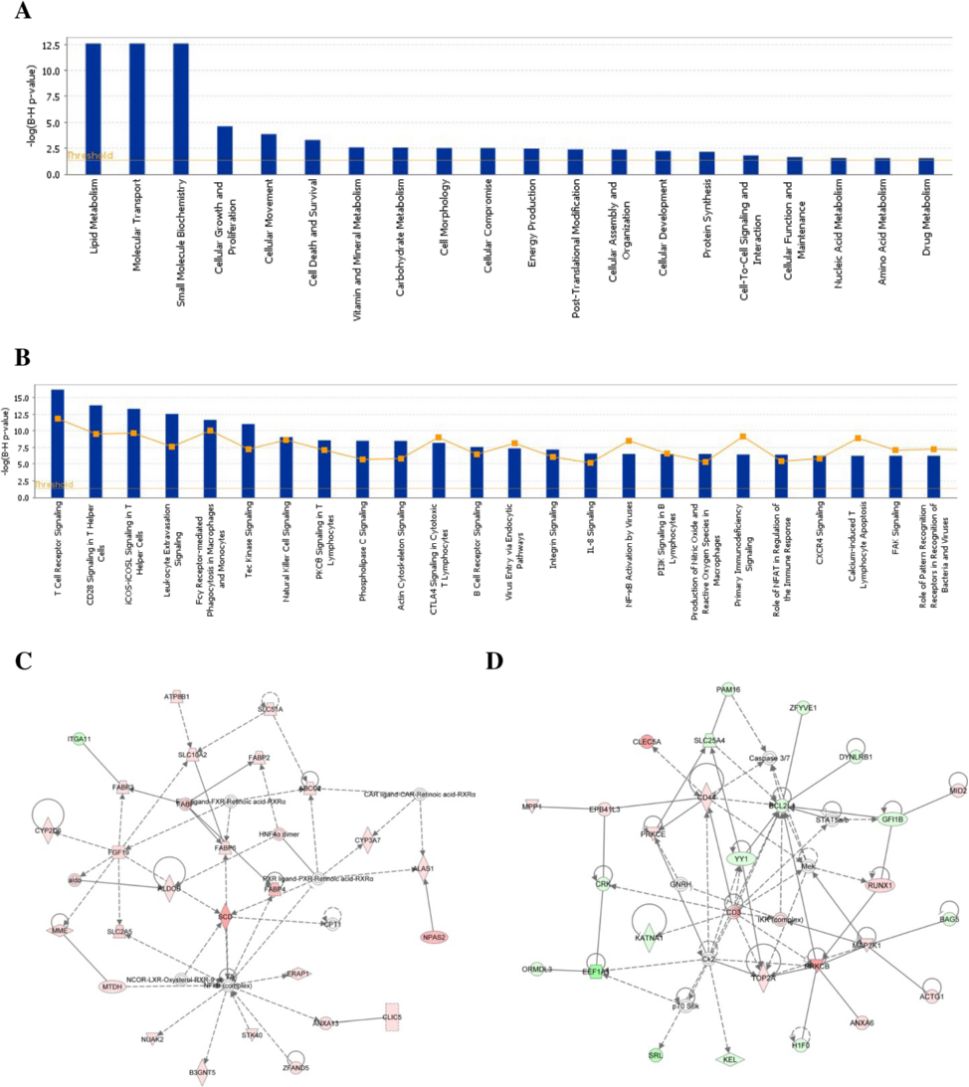
Ingenuity Pathway Analysis of the chicken response to HPAI infection in the ileum at 1 dpi and LPAI infection in the lung at 3 dpi. **a** Molecular functions of genes responding to HPAI in the ileum at 1dpi. **b** Biological pathways which are significantly altered during the host response to LPAI in the lung at 3dpi. In each case p < 0.05. **c** Differential gene regulation in a biological network concerned with lipid metabolism (ileum). **d** Genes differentially expressed in the cell death, cell signalling and inflammatory response network (lung). Up-regulated genes (red) and down-regulated genes (green). The deeper the colour, the higher the level of differential expression

The molecular function of the genes involved during the early response to H5N1 infection in the ileum is seen to be significantly represented by genes involved in lipid concentration and transport (Fig. 2a and c). Host cell lipids are important during influenza infection as they make up the bilayer of the virus carrying the haemagglutinin and neuraminidase glycoproteins and the matrix protein. Lipid rafts play an important role in the life cycle of the virus, including infection, assembly and budding. Lipid rafts are also important in the transport and assembly of viral components as well as in budding and virus release [34, 35]. The significance of the T- and B-cell response in the chicken can be seen during the host response to H5N2 infection in the lung at 3 dpi. Some of the most up-regulated genes include T-cell markers, CD8B, CD4, CD3E and CD28, along with *CD79B* which codes for part of the B-cell receptor complex and *CXCR5* which is involved in B-cell migration. Fig. 2b shows the canonical pathways significantly affected during the chicken host response to H5N2. One of the most perturbed biological networks (Fig. 2d) is that of cell death, cell signalling and the inflammatory response.

The data was also analysed for enrichment of biological parameters such as gene ontology (GO) terms, chromosomal location and the presence of transcription factor binding sites (Additional file 14: Figure S7). Examination of the genes up-regulated in response to H5N1 in the ileum at 1dpi shows a significant representation of genes involved in metabolic processes and lipid binding (*APOA4*, *KYNU*, *HNF4A*, *HSD17B4*, *FABP6*, *ANXA13*, *SNX24*, *SNX2*, *FABP4*, *FABP2*, *SPTLC1*, *ALAS1*, *HNF4β*, *APOA1*, and *FABP3)*. Genes being down-regulated are over-represented by genes involved in axon guidance, generation of neurons and in the regulation of neurogenesis (*TGFB2*, *EPHB2*, *SMO*, *SEMA3F*, *BMP7*, *CNTN4*, *CXCL12* and *EPHB3*). This effect on genes involved in neuron development confirms earlier reports of Alzheimer-like effects in mice infected with H5N1 [36] and possible association with Parkinsonism [37]. It also ties in with recent reports of brain damage caused by H7N9 in humans [38].

In response to H5N2 infection in the lung, significantly represented GO terms within up-regulated genes include protein binding, immune system processes, signal transduction, leukocyte activation, and cytokine binding. The up-regulated genes also show an enrichment of binding sites for the transcription factors *ELF1* and *ETV4*. ELF1 is known to be required for the T-cell-receptor-mediated trans-activation of HIV-2 gene expression. It also activates the *LYN* and *BLK* promoters. LYN and BLK both play important roles in the B-cell response. ETV4 is known to bind to the enhancer of the adenovirus E1A gene, activate matrix metalloproteinase genes and be associated with invasion and metastasis of tumour cells.

Interestingly, within the 3 chicken lung replicates which were infected with H5N2, only 2 samples showed the presence of virus when tested. All 3 samples showed a vigorous, tightly-correlated immune response. We therefore compared the 2 samples showing viral titre with the other sample, which was able to clear the virus. Clustering the data allowed us to highlight a particular group of genes which were more strongly expressed in the non-viral samples. Functional analysis of these genes showed them to be very immune-specific. Many of the genes were immunoglobulin genes and genes found on the surface of immune cells (particularly T-cells). This would indicate that these particular birds were able to prevent virus from actually entering the lung tissue by means of increasing expression of genes such as *PTPRC*, *CD44*, *CD3E*, *CD8B*, *CTLA4*, *TNFRSF13C*, *CD79B*, *IL2RG*, *CD4*, *ITGA4*, *CD5* and *CD28*.

### The duck host response to avian influenza infection

The response to influenza infection in the duck is seen to be quite different from that of the chicken. The low pathogenic H5N2 virus barely elicits much of an immune response at all with the peak number of genes expressing at 3 dpi in the ileum (which is still only 102 genes, FDR < 0.05 and fold change >1.5). This reflects how ducks are unperturbed by LPAI infection and suggests they may be able to somehow block the virus before it enters the cell. Upon infection with H5N1, the duck mounts a robust innate response, with large numbers of genes significantly differentially expressed in both lung and ileum. The response in the ileum increases from 1 to 3 dpi whereas in the lung, a large response is already underway 1 dpi and is maintained through 3 dpi. This differential response to H5N1 and H5N2 viruses confirms previous results by Vanderven et al. [2].

The biological pathways involved in the duck response to H5N1 infection (in the ileum at 1 dpi and in the lung at 3 dpi) were examined as described above for the chicken. During the early response to HPAI infection in the ileum, the TLR pathway is seen to be significantly activated (Additional file 15: Figure S8A). In the lung at 3 dpi, genes involved in the extra-cellular-matrix, focal adhesion, and adherens junctions are highly down-regulated, while genes involved in leukocyte transendothelial migration are up-regulated. Interestingly, genes associated with cancer-related processes are also seen to be activated/inhibited (Additional file 15: Figures S8B-D). Additional file 16: Tables S8A and B show all the pathways responding significantly in the duck ileum and lung respectively.

**Fig. 3:**
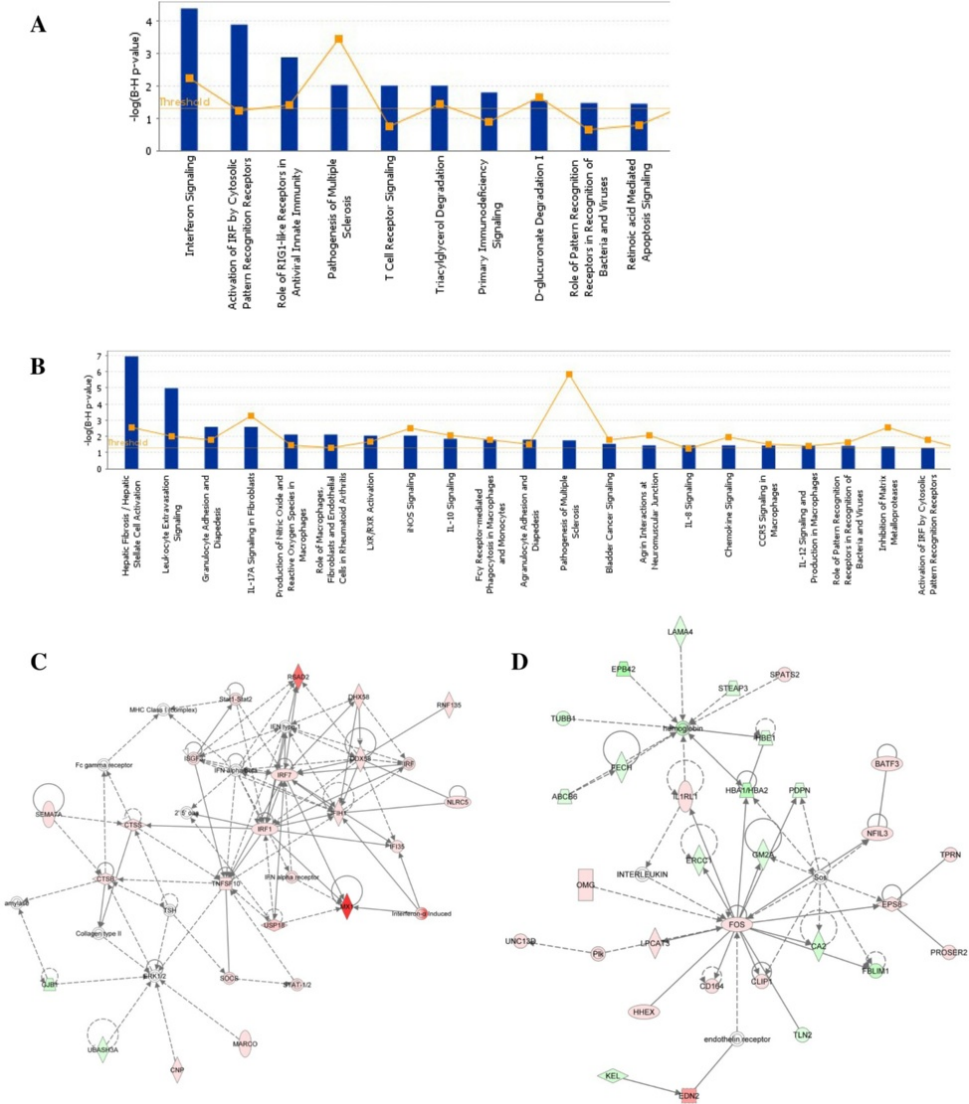
Ingenuity Pathway Analysis of the duck response to HPAI infection in the ileum at 1 dpi and in the lung at 3 dpi. **a** Biological pathways which are significantly altered during the host response to HPAI in the ileum at 1dpi. **b** Biological pathways which are significantly altered during the host response to HPAI in the lung at 3dpi. In each case p < 0.05. **c** Differential gene regulation in a biological network concerned with the inflammatory response network (ileum). **d** Differential expression of genes involved in haematopoiesis (lung). Red represents up-regulated genes and green down-regulated genes. The deeper the colour, the higher the level of differential expression

Pathway analysis also shows the effect of HPAI on hepatic stellate cells or lipocytes. The role of RIG-I (which is absent in the chicken genome) [18] is highlighted along with that of pattern recognition receptors (PRRs), indicating activation of *TLR1LA*, *TLR4* and *TLR7*. We have previously shown TLR1LA to be activated during the host response to Marek’s Disease Virus [39]. Levels of TLR4 are increased by heat shock proteins and various cellular factors produced during infection, while the ligand for TLR7 is ssRNA, of which the H5N1 genome is comprised. IRF, IL8, IL10 and IL17A signalling are all also up-regulated, indicating a robust cytokine response (Fig. 3a and b). Some of the most highly up-regulated genes seen during the duck host response to H5N1 include *RSAD2* (viperin) which encodes an interferon-inducible antiviral protein involved in TLR7-dependent production of IFNB, *MX1* which is known to have antiviral activity against avian influenza and *DDX58* (*RIG-I*) which encodes a pattern recognition receptor for viruses. The two main biological networks observed in the duck host response are those concerned with inflammatory response (1 dpi in the ileum, Fig. 3c) and haematopoiesis (3 dpi in the lung, Fig. 3d).

Enrichment analysis of the genes involved in the duck response to H5N1 (Additional file 17: Figure S9) shows that the over-represented GO terms within up-regulated genes include signal transduction, response to stimulus, and cell communication while the down-regulated genes represent those involved in ion binding and cell structural organization. Examination of chromosomal location indicated a strong representation of genes from Chr1 amongst those up-regulated. An enrichment of the NF-κB TFBS was also identified within the up-regulated genes. NF-κB is known to have a central role to regulation of the immune response.

### Comparison of chicken and duck host responses to LPAI and HPAI viruses

**Fig. 4:**
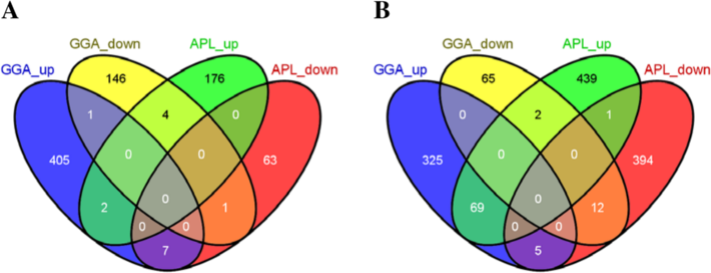
Venn diagram showing shared and unique responses to AI infection in duck and chicken. **a** Chicken response to H5N1 infection in the ileum at 1 dpi is compared to that of the duck. **b** Chicken response to H5N2 infection in the lung at 3 dpi is compared with the duck response to high path infection in the lung at 3 dpi

In the ileum at 1 dpi, both chickens and ducks mounted a fairly large response to infection with H5N1. In chicken there were 403 unique, annotated genes which were up-regulated in response to H5N1 and 147 genes which were down regulated (this represents 86 % of all genes with a FC >1.5 and 97 % of all annotated genes). In duck there were 174 unique, annotated genes up-regulated in response to H5N1 and 63 genes down-regulated (representing 74 % of all genes with a FC >1.5 and 94 % of all annotated genes). Fig. 4a shows how these genes overlap in each response. Only 2 genes were commonly up-regulated in both duck and chicken (CYP2D6, TDRD7), and only 1 commonly down-regulated (NPTX2).

With the largest immune response in the lung being against H5N2 in chicken and against H5N1 in duck, it was decided to compare the genes being expressed in the lung at 3 dpi for each infection scenario. In chicken there were 323 unique, annotated genes which were up-regulated in response to H5N2 and 65 genes which were down regulated (this represents 70 % of all genes with a FC >1.5 and 81 % of all annotated genes). In duck there were 430 unique, annotated genes up-regulated in response to H5N1 and 394 genes down-regulated (representing 74 % of all genes with a FC >1.5 and 90 % of all annotated genes). Fig. 4b shows how these genes overlap in each response. As can be seen, only 68 genes were commonly up-regulated in both duck and chicken and only 12 commonly down-regulated. Both these comparisons show that the largest part of the early response to influenza infection is unique to each species.

The genes responding uniquely in each host were examined further. Comparing the host-specific responses to H5N1 infection in the ileum at 1 dpi we see that the chicken response focuses on B-cell activation and lipid metabolism. The duck, on the other hand, is expressing genes involved in the differentiation of T-cells, cell death, and the activation of interferon’s and cytokines. In the lung at 3 dpi, the unique chicken response to H5N2 is overwhelmingly concerned with T- and B-cell development and activation and cell death, whereas the genes expressed uniquely in duck upon H5N1 infection are all concerned with pathogen-associated molecular patterns and the RIG-I and TLR pathways (Additional file 18: Figure S10).

### Gene expression profiles of *IFITM* subfamily members suggest specific roles in host responses towards avian influenza infections

Availability of transcriptomic data now allowed investigation of the *IFITM* gene expression response during LPAI and HPAI infection in both duck and chicken. Study of *IFITM* gene expression after both H5N1 and H5N2 infection has allowed us to see a completely different host response in each species (Table 3). In chicken there is a very limited *IFITM* response. The only gene expression seen is in the ileum 1 dpi after H5N1 infection, where *IFITM1* is seen to decrease slightly (1.6-fold) and *IFITM3* increases 2-fold. The duck, on the other hand, is seen to mount a robust IFITM reaction to influenza infection. In response to low path infection, the duck increases expression of both *IFITM1* and *2* (~3-fold) in both lung and ileum. An early response at 1 dpi is obviously sufficient, with expression seen to diminish by 3 dpi. The duck is also clearly able to raise a strong interferon response against the highly pathogenic virus, with large increases in expression seen from *IFITM1* and *2* in both lung and ileum. There are also very large increases in expression of *IFITM3* (up to 93-fold) in lung.

**Table 3:** Expression of IFITM genes in response to AI infection in chicken and in duck. Numbers refer to fold-change in gene expression

A. CHICKEN								
	Low path infection				High path infection			
	Ileum		Lung		Ileum		Lung	
Gene	Day 1	Day 3	Day 1	Day 3	Day 1	Day 3	Day 1	Day 3
IFITM1					↓2			
IFITM2								
IFITM3					↑2			
B. DUCK								
	Low path infection				High path infection			
	Ileum		Lung		Ileum		Lung	
Gene	Day 1	Day 3	Day 1	Day 3	Day 1	Day 3	Day 1	Day 3
IFITM1	↑3	↑3	↑3		↑10	↑6	↑12	↑4
IFITM2	↑4		↑3		↑13	↑5	↑27	↑6
IFITM3							↑93	↑24

**Fig. 5:**
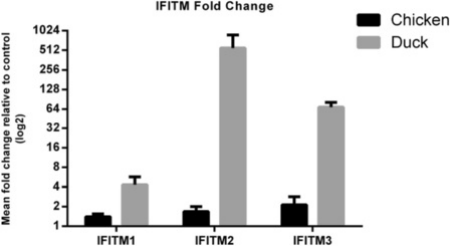
QRT-PCR analysis of lung RNA at 1dpi (H5N1) in chicken and duck. IFITM gene expression in lung tissue measured by qRT-PCR in control and HPAI H5N1 (A/Vietnam/1203/04) infected chicken and duck samples 1 dpi. IFITM gene expression was measured in three control and three infected birds. Data are expressed as the mean fold change in infected birds relative to the uninfected controls. Error bars represent the standard deviation

To confirm these differences in gene expression seen between the species, QRT-PCR experiments were performed using RNA samples from the lungs of birds at 1 day post H5N1 infection (when the largest differences are seen). Samples from 3 infected birds were compared to 3 uninfected controls and normalized against eukaryotic 18 s rRNA expression. Large differences in *IFITM* expression were confirmed, with virtually no response in the chicken and significant up-regulation in the duck (Table 4 and Fig. 5). The very large *IFITM2* response seen in the duck (553-fold up-regulation) is most probably an over-exaggerated estimate due to the wide variability seen in uninfected ducks (Additional file 19: Figure S11). If the outlier bird is removed, then up-regulation is approximately 16-fold, which is similar to the relative ratios of expression seen with the RNAseq data.

**Table 4:** QRT-PCR analysis of the IFITM genes in chicken and duck

	IFITM1			IFITM2			IFITM3		
	Mean fold change	Mean ΔCt	SD ΔCt	Mean fold change	Mean ΔCt	SD ΔCt	Mean fold change	Mean ΔCt	SD ΔCt
Infected Duck	4.3	6.2	0.4	553.1	6.4	0.7	68.9	11.7	0.5
Infected Chicken	1.4	7.4	0.1	1.7	7.6	0.2	2.1	18.5	0.2

**Fig. 6:**
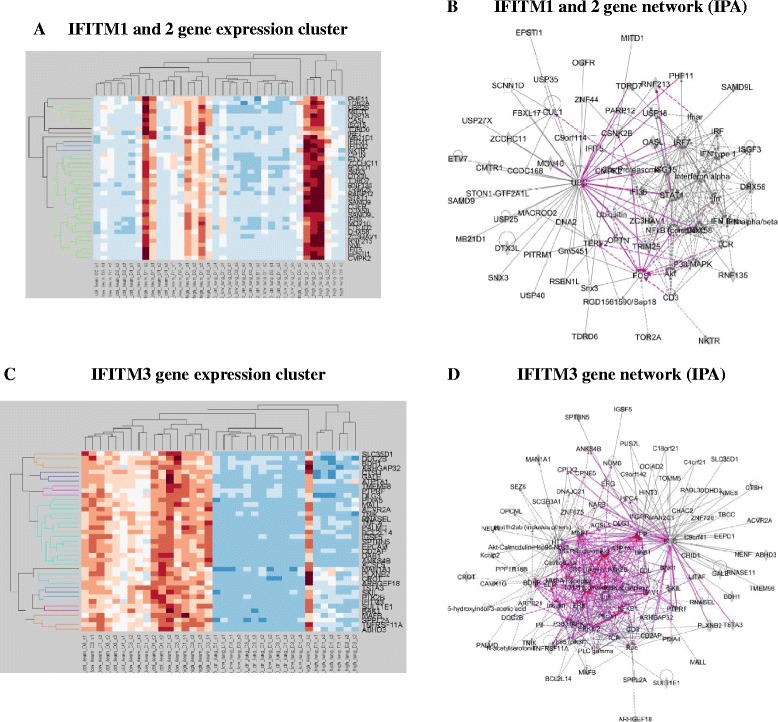
Co-expression clusters associated with the duck *IFITM1, 2* and *3* genes. **a** *IFITM1* and *2* gene expression cluster, **b** IFITM1 and 2 network, **c** *IFITM3* gene expression cluster, **d** IFITM3 network. The gene expression clusters were calculated using CLUSTERGRAM and normalised data from all the RNAseq datasets. The gene networks were calculated using IPA analysis of the genes that were clustered with expression of IFITM genes in **a** and **c**

In order to examine the potential role of the avian *IFITM* genes in the global host response to avian influenza from LPAI and HPAI viruses the expression data (from duck) was clustered using CLUSTERGRAM (Fig. 6) and the functional profile of genes grouping with *IFITM1*, *2* and *3* were examined using DAVID and IPA (for details see *Materials and Methods*). In this way we could identify genes that are co-regulated with the *IFITM* genes and thus infer correlated gene functions (“guilt by association”). Avian *IFITM1* and *2* were seen to cluster with genes highly enriched with roles in anti-viral responses (Additional file 20: Table S9) while IFITM3 was very different and clustered with genes involved with organelle membranes and apoptotic processes (Additional file 21: Table S10). Avian *IFITM3* is also co-expressed with genes involved in phosphorylation. Interestingly, phosphorylation of IFITM3 is thought to be important in determining its sub-cellular location and anti-viral function [40]. The results of this study confirm the involvement of avian IFITM1, 2 and 3 in the host response to avian influenza LPAI and HPAI viruses.

### Structural, functional and evolutionary constraints on amino acid residues within chicken, duck and human IR-IFITM proteins

**Fig. 7:**
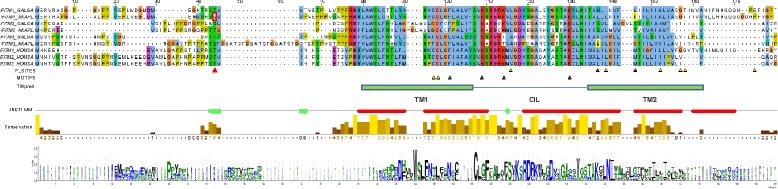
Domain analysis and sequence characteristics of the IFITM1, 2 and 3 gene family. The sequence alignment of chicken (GALGA), duck (ANAPL) and human (HOMSA) IFITM peptides was created using T-coffee and displayed using Jalview, which was also used to create secondary structure (JNETHMM; red, helices; green, beta sheets) and conservation tracks. Sites predicted to be under positive selection (P_SITES, Table 1) are shown as triangles (red, duck and yellow, other birds). The transmembrane (TM1 and TM2), conserved intracellular loop (CIL) and other domains were predicted using SMART, SOSUI and ExPasy. MOTIFS, mark amino acid residues as triangles and are discussed in the main text: yellow Cysteine, green Phenylalanine, purple Lysine. Sequence logos were generated using Weblogo and based on the alignment of the duck, chicken and human IFITM sequences. For details of methods see Materials and Methods

The gene expression results suggest that the avian IFITM1, 2 and 3 proteins play a role in the host’s antiviral response towards avian influenza virus strains. The phylogenetic analysis revealed codons under positive selection in the *IFITM1* and *3* genes. This would suggest that these proteins may have been subjected to selection pressure from pathogen infection, possibly ssRNA viruses like avian influenza. Multiple amino acid sequence alignments of the proteins encoded by these avian *IFITM1* and *3* genes reveals a number of conserved motifs, shown to be functional in the homologous regions of the human IR-IFITM proteins (Fig. 7). All these proteins share the highly conserved TM1-CIL-TM2 structure, core to the IFITM protein family. The TM1-CIL or CD225 domain is the most conserved region. The TM2 domain is more variable with most of the sites under pervasive or episodic positive selection mapping to this region. This domain is likely to recognise lipid motifs on the surface of viral and other pathogens.

The alignment also reveals that other functionally significant amino acids are conserved in some or all of the avian IFITM1, 2 and 3 proteins. In huIFITM3 conserved cysteines (C71, 72 and 105) are palmitoylated and are required for viral restriction [41]. Mutagenesis studies of these cysteines in huIFITM3 [26] show they are required for protein clustering in membranes and antiviral activity. Only the cysteine equivalent to huIFITM3 C72 (C98 in Fig. 7) is conserved in all avian IFITM1, 2 and 3 proteins, which is likely to be the core palmitoylation site required for membrane position. The more N-terminal cysteine C71 in huIFITM3 (C97 in Fig. 7) is only conserved in avian IFITM1 but not IFITM2 or 3.

In huIFITM3 the four lysine residues in the N-terminal and CIL domains are ubiquinated, control IFITM3 protein degradation and antiviral activity [42, 43]. Two of these lysine residues, equivalent to huIFITM3 K83 and 104 (K109 and 130, in Fig. 7) are conserved in avian IFITM1, 2 and 3 proteins. The third lysine equivalent to huIFITM3 K88 is conserved in avian IFITM1 and 2, but not 3.

The N- and C-termini are poorly conserved and of variable length in both birds and mammals. In huIFITM2 and 3, a conserved tyrosine (Y20) in the N-terminal domain may determine cellular location [42], with huIFITM1 mostly at the plasma membrane and huIFITM2 and 3 located mostly in intracellular compartments. In birds this tyrosine (Y20 in Fig. 7) is conserved in avian IFITM1 and 3, but not IFITM2. This would predict an intracellular location for avian IFITM1 and 3, and plasma membrane for IFITM2 proteins.

John et al. [26] has shown that huIFITM3 can interact with IFITM1 and 2 and itself through specific phenylalanine residues (F75 and F78). The formation of these homo- and hetero-oligomers may determine their cellular location [26]. The N-terminal phenylalanine equivalent to huIFITM3 F75 in TM1 is conserved in birds (F101 in Fig. 7) in most IFITM1, 2 and 3 proteins, except chIFITM3. A non-conserved leucine (L101 in Fig. 7) is found in chIFITM3 and may have functional consequences, which remains to be tested. Finally, valine 30 (V44 in Fig. 7) is under positive selection in the duck and is located in a region known to restrict influenza virus entry in huIFITM3 [26].

## Discussion

The elucidation of why ducks and chickens show very different tolerances to avian influenza infection will help underpin research into prevention of the economic damage to the poultry industry and a potentially devastating human pandemic. Investigation of the host responses to infection in these species thus has important implications for not only avian well-being, but also human health. In this study we use transcriptomic sequencing to analyse gene expression differences in the two avian species. We show very different *in vivo IFITM* responses in ducks and chickens matched by differences in selection pressures and evolutionary history of the *IFITM* gene family.

Identification of *IFITM* genes in various avian species has allowed us to compare the pattern of evolution of these genes in birds with that of mammals. Interestingly, two completely different evolutionary patterns seem to have taken place within the two groups. In mammals the *IFITM1*, *2* and *3* genes tend to cluster together within species-specific lineages, suggesting independent gene duplication events within species to create paralogous genes. In birds, however, each member of the IFITM family clusters into an independent clade (Fig. 1). We have shown that these genes are rapidly evolving and highlighted particular amino acids which appear to be under positive selection in certain species. Amongst the bird species, we also see that *IFITM1* appears to be evolving more rapidly in the duck lineage.

Transcriptomic sequencing of chicken and duck tissues infected with H5N1 and H5N2 influenza viruses has allowed us insight into differences in the host immune response in each species and provided us with information on the different biological pathways which are induced in each response. The chicken immune system is able to counter LPAI H5N2, but unable to respond effectively to the HPAI H5N1. B- and T-cells are induced in response to H5N2, but an initial early immune response in the ileum against H5N1 is ultimately insufficient to clear the virus and the animal succumbs to infection. The response seen in the ileum primarily involves lipid metabolism genes. The duck, on the other hand, is barely required to produce a response against H5N2 since LPAI is not seen as pathogenic and is able to initiate a robust host response to H5N1 infection. A strong cytokine response along with induction of the TLR and RIG-I pathways ensure that the duck is able to survive or delay infection with the more pathogenic H5N1 virus. RIG-I is involved in the initial cytosolic detection of virus in the host cell and TLRs are responsible for the induction of signalling cascades leading to the production of type-I interferons. When the chicken response to H5N2 was compared to the H5N1 response in the duck, two unique host-specific responses were seen.

Enrichment analysis of differentially expressed genes showed that genes involved in protein binding and various immune-associated processes were highly represented in the duck. The duck host response to H5N1 also showed the down-regulation of many genes involved with metabolic systems and metal-ion binding. In the chicken ileum response to H5N1 at 1 dpi, many genes involved in lipid metabolism and binding are also up-regulated, while neuronal-associated genes were notably down-regulated. The effect on neural processes during influenza infection has been reported in recent studies [36–38]. Analysis of the presence of transcription factor binding sites amongst down-regulated genes highlighted an enrichment of genes with binding sites for ELF1 (implicated in T-cell responses and lipid metabolism) and ETV4 (involved in B-cell responses) in chicken.

Specific examination of the role of the *IFITM* genes during avian influenza infection was also made. IFITM1, 2 and 3-like proteins are already known to have anti-viral function in mammals [23], with IFITM3 specifically shown to restrict the effects of influenza in a mouse knockout model [20]. The sequence data presented in this study was therefore used to determine their activity in birds. A recent report by Smith et al. [28] demonstrates that over-expression of chicken *IFITM1* (annotated as *chIFITM3* in that study) restricts influenza *in vitro*. We show that *in vivo* chickens mount a nearly non-existent *IFITM* response, with an ineffectual early induction of *IFITM3* in the ileum against H5N1 infection, which is not maintained. Conversely, the duck is able to mount and sustain an effective induction of *IFITM1*, *2* and *3* in both the lung and ileum during H5N1 progression. Chickens are seen to show an early up-regulation of lipid metabolism genes in the ileum against HPAI. This is most likely a reflection of the viral/host membrane fusion and subsequent viral replication which is occurring. This lipid response is not seen in the duck. IFITM1, 2 and 3 have been shown to block viral membrane hemifusion (when the outer membranes of the two lipid bilayers have fused but the inner membranes are still intact). The IFITM proteins alter the lipid composition of the membrane and decrease membrane fluidity, thus restricting viral fusion [44]. So, the differential response of lipid-associated genes seen between chicken and duck is most probably due, in part, to the hugely different IFITM response seen between these two species.

## Conclusions

Along with the presence/absence of various immune genes and the differing affinities of the influenza virus for host sialic acid receptors, the different *IFITM* responses we have seen in chickens and ducks are very probably, one of the reasons contributing to the duck’s ability to mitigate HPAI where it proves lethal to the chicken. The evolutionary selective forces also acting upon the *IFITM* genes will, in turn, promote mutation of the influenza viruses themselves as part of the on-going ‘arms race’ of which viruses and hosts find themselves a part. The apparent absence of *RIG-I* in Galliform birds [17] along with the poor *IFITM* expression we have seen during infection in chickens may also be contributory factors as to why certain H5 and H7 viral subtypes are able to become highly pathogenic in these birds. Why only H5 and H7 strains (thus far) are evolving in their pathogenic potential remains to be determined. The emergence of the H7N9 influenza subtype and its recent infection of humans, along with the developing susceptibility of ducks to some highly pathogenic H5N1 viruses [4] highlight the ever-increasing need to identify and understand the resistance mechanisms deployed by hosts against these viruses.

## Methods

### Characterisation of chicken and duck interferon-induced transmembrane (IFITM) protein genes

The location of *IFITM*-like gene sequences in the chicken (Galgal4; GCA_000002315.2) and duck (BGI_duck_1.0; GCA_000355885.1) genomes were initially defined from blast [45] homology searches and the current Ensembl gene annotations. Genewise [46] was used to predict coding regions in genomic DNAs using avian or mammalian IFITM peptide sequences. In some cases 5’-RACE was used to determine the full coding sequence from total RNA isolated from spleen tissues. 5'-RACE was performed on 10 μg of duck spleen total RNA using the First Choice RLM-RACE kit (Invitrogen). Briefly, the RNA was treated with Calf Intestinal Phosphatase to remove 5'-phosphates from degraded mRNA, rRNA, tRNA and DNA. The cap structure on intact mRNA was removed with Tobacco Acid Pyrophosphatase allowing a proprietary 5'-RACE adapter to be ligated to the de-capped mRNA. Random-primed reverse transcription followed by nested PCR, with supplied forward primers and gene specific reverse primers (Additional file 22: Table S11), was performed to amplify the 5' ends of the *IFITM* genes. PCR conditions: 1st round: 1 μl of the RT reaction was amplified with 10pmol of each outer primer in 20 μl using FastStart Taq polymerase (Roche) in the supplied reaction buffer containing 200 μm each dNTP and 1x GC-RICH solution (Roche). Amplification conditions were 5 min at 95 °C followed by 35 cycles of 30 s at 95 °C, 30 s at 60 °C, 1 min at 72 °C, followed by 5 min at 72 °C. 2nd round: 2 μl of the first round PCR was amplified with 25pmol of each inner primer in 50 μl using FastStart Taq polymerase (Roche) in the supplied reaction buffer containing 200 μm each dNTP and 1x GC-RICH solution (Roche). Amplification conditions were 5 min at 95 °C followed by 35 cycles of 30 s at 95 °C, 30 s at 60 °C, 1 min at 72 °C, followed by 5 min at 72 °C. Duck sequences have been submitted to the public databases under the following accession numbers: IFITM1: GenBank- KF584226; IFITM2: GenBank- KF584227; IFITM3: GenBank- KF584228; IFITM5: EMBL- HG764554; IFITM10: GenBank- KF584229.

### Structural analyses of *IFITM* peptide sequences

The presence of conserved domains (e.g. CD225), transmembrane domains and other motifs (e.g. N-linked glycosylation sites) in the IFITM proteins were predicted using SMART [47] and SOSUI [48] and ExPasy [49], respectively. T-coffee [50] was used for multiple sequence alignment of peptide sequences and viewed using Jalview [51]. Weblogo was used to display consensus logos peptide sequences [52].

### Molecular phylogenetic analyses

IFITM coding and peptide sequences were obtained from GenBank [53], Ensembl [54] and as part of the Avian Phylogenomic Project hosted by Beijing Genomics Institute [55] as detailed in Additional file 2: Table S1. Multiple sequence alignments were performed using MUSCLE [56]. Bayesian inference trees were reconstructed using MrBayes v3.2.2 [57]. Peptide sequences of IFITM1, 2 and 3 proteins were analysed, set with priors (prset aamodelpr = mixed) and substitution model (lset rates = invgamma Ngammacat = 8). Four independent Markov Chain Monte Carlo (MCMC) chains were used with the temperature of 0.2. Two repetitions were run for 1,000,000 generations with tree and parameter sampling occurring every 1,000 generations. The first 25 % of trees were discarded as burn-in, leaving 750 trees per run. Posterior probabilities for internal node were calculated from the posterior density of trees. To confirm the general conclusions from MrBayes analysis we also used Maximum parsimony (MP), Neighbour-Joining (NJ) and Maximum likelihood (ML) to reconstruct trees with bootstrap values of 500 repetitions within the MEGA6 package [58]. Phylogenetic trees were displayed using FigTree [59].

### Positive selection analyses

First we used two methods implemented in DATAMONKEY [60] for detecting positive selection: the “Mixed Effects Model of Evolution” (MEME) and the “Fast Unbiased Bayesian Approximation” (FUBAR). MEME can find evidence of both episodic and pervasive positive selection at individual codons, even when the majority of branches are subject to purifying selection. FUBAR on the other hand was designed to detect pervasive positive selection at individual codons. Therefore the additional sites detected by MEME and not by FUBAR are likely to have been subject to episodes of positive selection. For these analyses the best fitting nucleotide substitution model was determined through the automatic model selection tool available on the server. To investigate whether gene conversion occurred in avian *IFITM* genes all sequences were screened for recombination using GARD implemented within the DATAMONKEY server. No evidence of recombination was found in these avian sequences. We also compared the results from DATAMONKEY with two alternative models implemented in CODEML (PAML 4.4) [61]: M8, which allows for codons to evolve under positive selection (dN/dS > 1) and M8A, which does not (dN/dS ≤ 1). These two nested models were compared using a likelihood ratio test (LRT) with two degrees of freedom. The analysis was run several times with the F3x4 model of codon frequencies. Codons under positive selection for model M8 were identified using a Bayes Empirical Bayes (BEB) approach and considering a posterior probability of > 95 %. For each analysis the species tree used was based on a new phylogenetic tree of birds based on evidence from the multiple sequence alignment of 48 avian genomes [62] as part of the “Bird Phylogenomic Project”, hosted by the Beijing Genomics Institute [55].

### Virus strains

A/Mallard/British Columbia/500/2005 (H5N2) was used for LPAI challenge. A/Vietnam/1203/2004 Clade 1 (H5N1) was used for HPAI challenge. The viruses were grown in the allantoic cavities of 10-day-old embryonated chicken eggs for 48 h at 35 °C. Allantoic fluid containing virus was harvested and stored at −80 °C until use. Virus yield was determined as 50 % egg infectious dose (EID_50_) per millilitre of virus stock by the method of Reed and Muench [63].

### Experimental animals

Specific pathogen–free white leghorn chickens were purchased from Charles River Laboratories (North Franklin, CT). Domestic Gray Mallards were purchased from Ideal Poultry (Cameron, TX). All experiments involving animals were approved by the Animal Care and Use Committee of St. Jude Children’s Research Hospital and performed in compliance with relevant policies of the National Institutes of Health and the Animal Welfare Act. All animal challenge experiments were performed in an animal biosafety level 2 containment facilities for the LPAI challenges and in biosafety level 3 enhanced containment laboratories for the HPAI challenges.

### Viral challenge

20 Chickens and 20 ducks, except the mock infection control group of 5 chickens and 7 ducks, were challenged with 10^6^ EID_50_ intranasally, intratracheally, and intraocularly of LPAI A/Mallard/British Columbia/500/2005 (H5N2) in phosphate buffered saline (PBS). 20 chickens, except the mock infection control group of 5 chickens, were challenged with 10^1.5^ EID_50_ intranasally, intratracheally, and intraocularly of HPAI A/Vietnam/1203/2004 (H5N1) in PBS. 20 ducks, except the mock infection control group of 8 ducks, were challenged with 10^6^ EID_50_ intranasally, intratracheally, and intraocularly of HPAI A/Vietnam/1203/2004 (H5N1) in PBS. During LPAI and HPAI infections in chickens and ducks, mock infection control groups were also inoculated. Mock infection control birds received an equivalent volume and route of administration with PBS. Animals were monitored daily for clinical signs.

### Tissue sampling

Lung and ileum samples were collected from all birds on days 1 and 3 dpi. Tissue homogenates were inoculated into 10-day-old embryonated chicken eggs in triplicate to screen for positive samples. Positive samples were then titrated in eggs and expressed as log_10_EID_50_/mL as described by Reed and Muench [63]. The lower limit of detection was 0.75 log_10_EID_50_/mL. RNA extraction: For each ileum and lung tissue, the tissue sample was homogenized in Trizol (Invitrogen Life Technologies), 0.1 g tissue/1.0 ml Trizol with handheld homogenizer Power Gen125 (Thermo Fisher Scientific). After homogenates were prepared, the samples were centrifuged to separate the liquid phases of Trizol homogenates. 0.5 ml of the aqueous phase was passed through a QIagen RNeasy (Qiagen, Valencia, CA) spin column according to manufacturer’s instructions for RNA extraction. Samples were frozen and stored at −80 °C until all samples were collected for analysis.

### RNA Sequencing

Samples were prepared for mRNA sequencing using 5ug of total RNA starting material following the Illumina mRNA sequencing 8 sample preparation kit protocol. Resulting libraries were quality checked on an Agilent DNA 1000 bioanalyzer (Agilent Technologies, South Queensferry, UK) and then clustered onto a Single Read flowcell using the Illumina v2 cluster generation kit at a 4.75pM concentration. Thirty-six cycle single-ended sequencing was carried out on the Genome Analyser IIx using Illumina v3 Sequencing by Synthesis kits (Illumina, Little Chesterford, UK).

### Processing of next generation transcriptome sequencing

Ileum and lung RNAs were analysed from PBS infected control (3 samples from each of 1dpi and 3dpi), H5N1-infected (3 samples from each of 1dpi and 3dpi) and H5N2-infected (3 samples from each of 1dpi and 3dpi) chickens and ducks. The Illumina Genome Analyzer-IIx platform generated millions of RNAseq tags per sample (255 million in total for chicken, 286 million for duck), each 36 nucleotides in length. RNAseq tags were processed in several steps: (1) tags were filtered using quality scores, (2) tags were assigned to a region of the reference genomes (Galgal4 and BGI_duck_1.0 [14] by parallel computing using SOAP2, (3) tags were cross referenced to Ensembl (Genebuild72) gene annotations and (4) counts of tags were calculated for each gene. Based on this pipeline, 231 million tags were mapped to the chicken genome, 151 million of which were annotated to known genes in the chicken (65 %). 239 million duck sequence tags could be mapped to the duck genome, 133 million of which were annotated to known genes in the duck (56 %). Statistical analysis was then used to identify differentially expressed genes (using the edgeR package within Bioconductor; FDR < 0.05 and FC > 1.5). Sequences have been submitted to Array Express under accession numbers E-MTAB-2908 (chicken data) and E-MTAB-2909 (duck data).

### Quantitative reverse transcription polymerase chain reaction (QRT-PCR)

Approximately 3 μg of total RNA was reverse transcribed to produce cDNA using random hexamers and AffinityScript multi Temp cDNA kit (Agilent Technologies). Quantitative PCR primers for all chicken and duck IFITM genes were designed using Custom Plus Taqman® RNA Assays Design tool (Applied Biosystems®). The Taqman® endogenous control gene Eukaryotic 18S rRNA was used as a singleplex control (Applied Biosystems®). The qRT-PCR reactions were performed according to manufacturer’s instructions using approximately 50 ng cDNA per reaction and Taqman® universal PCR master mix, no AmpErase® UNG (Applied Biosystems®). The reactions were run on an Applied Biosystems® 7500 Real-Time PCR machine and the data were analysed using the ddCt method [64] on 7500 software version 2.0.6.

### Bioinformatics analyses

In order to determine which biological pathways are involved in the responses to viral infection, Pathway Express [65] was used. Genes differentially expressed during the host response (False discovery rate (FDR) <0.05) were analysed against a reference background consisting of all genes expressed in the experiment. Annotation of the chicken genes was based upon the human orthologs. Use of the IPA program (Ingenuity® Systems) [66] revealed which canonical pathways and physiological functions are affected by AIV infection in the host (Benjamini-Hochberg multiple testing correction; FDR < 0.05). Genes were clustered by similar expression pattern and analysed for enriched GO-terms and transcription factor binding sites (TFBS) using Expander (v5.1) [67]. Normalised expression data from control samples were compared with infected samples to examine the host response to AIV infection. Enrichment of particular GO terms or TFBS within clusters was done by using the TANGO and PRIMA algorithms, respectively, within the Expander package. Analysis of genes clustering with the IFITM genes was carried out by first grouping the genes using “clustergram” within the MATLAB Statistics toolbox (2009) [68] and then examining their function using DAVID v6.7 [69, 70].

## Additional files

Additional file 1: Figure S1. Domain structure of the chicken and duck IFITM proteins as determined by the SMART algorithm [47]. Blue blocks show the transmembrane regions while pink defines areas of low complexity. The resultant coding sequences were as follows: *IFITM1* - 420 nucleotides (140 amino acids); *IFITM2* - 327 nucleotides (109 amino acids); *IFITM3* - 429 nucleotides (143 amino acids); *IFITM5* - 402 nucleotides (134 amino acids); *IFITM10* - 591 nucleotides (197 amino acids). (PPTX 84 kb) Additional file 2: Table S1. Source of IFITM protein sequences used in phylogenetic analyses. (XLSX 16 kb) Additional file 3: Table S2. Species names and abbreviations used in phylogenetic analyses. (XLSX 16 kb) Additional file 4: Figure S2. Multiple sequence alignment of IFITM proteins using MUSCLE. (TXT 64 kb) Additional file 5: Figure S3. Molecular Phylogenetic analysis by Maximum Likelihood method of IFIT proteins using MSA in Additional file 4: Figure S2. The evolutionary history was inferred by using the Maximum Likelihood method based on the JTT matrix-based model. The bootstrap consensus tree inferred from 1000 replicates is taken to represent the evolutionary history of the taxa analysed. Branches corresponding to partitions reproduced in less than 50 % bootstrap replicates are collapsed. The percentage of replicate trees in which the associated taxa clustered together in the bootstrap test (1000 replicates) are shown next to the branches. Initial tree(s) for the heuristic search were obtained automatically by applying Neighbor-Join and BioNJ algorithms to a matrix of pairwise distances estimated using a JTT model, and then selecting the topology with superior log likelihood value. A discrete Gamma distribution was used to model evolutionary rate differences among sites (5 categories (+G, parameter = 3.0096)). The analysis involved 205 amino acid sequences. All positions with less than 95 % site coverage were eliminated. That is, fewer than 5 % alignment gaps, missing data, and ambiguous bases were allowed at any position. There were a total of 98 positions in the final dataset. Evolutionary analyses were conducted in MEGA6. (PPTX 226 kb) Additional file 6: Figure S4. Multiple sequence alignment of IFITM1, 2 and 3-like proteins using MUSCLE. The transmembrane domains and other features are shown at the foot of the figure, and were displayed using options from Jalview. For more details see legend to Fig. 6. (PPTX 801 kb) Additional file 7: Figure S5. Evolutionary relationships of IFITM1, 2 and 3 proteins in vertebrates. (A) The evolutionary history was inferred using the Maximum Parsimony method (PAUP). The bootstrap consensus tree inferred from 500 replicates is taken to represent the evolutionary history of the taxa analysed. Branches corresponding to partitions reproduced in less than 50 % bootstrap replicates are collapsed. The percentage of replicate trees in which the associated taxa clustered together in the bootstrap test (500 replicates) are shown next to the branches. The MP tree was obtained using the Subtree-Pruning-Regrafting (SPR) algorithm with search level 1 in which the initial trees were obtained by the random addition of sequences (10 replicates). The analysis involved 148 amino acid sequences. All positions with less than 95 % site coverage were eliminated. That is, fewer than 5 % alignment gaps, missing data, and ambiguous bases were allowed at any position. There were a total of 86 positions in the final dataset. Evolutionary analyses were conducted in MEGA6. (B) The evolutionary history was inferred using the Neighbour-Joining method (NJ). The bootstrap consensus tree inferred from 500 replicates is taken to represent the evolutionary history of the taxa analysed. Branches corresponding to partitions reproduced in less than 50 % bootstrap replicates are collapsed. The percentage of replicate trees in which the associated taxa clustered together in the bootstrap test (500 replicates) are shown next to the branches. The evolutionary distances were computed using the JTT matrix-based method and are in the units of the number of amino acid substitutions per site. The rate variation among sites was modelled with a gamma distribution (shape parameter = 5). The analysis involved 148 amino acid sequences. All positions with less than 95 % site coverage were eliminated. That is, fewer than 5 % alignment gaps, missing data, and ambiguous bases were allowed at any position. There were a total of 86 positions in the final dataset. Evolutionary analyses were conducted in MEGA6. (C) The evolutionary history was inferred by using the Maximum Likelihood method (ML). The bootstrap consensus tree inferred from 500 replicates is taken to represent the evolutionary history of the taxa analysed. For more details see legend to Additional file 5: Figure S3. (D) Bayesian tree of the vertebrate IFITM1, 2 and 3-like gene family calculated using MrBayes. For more details see legend to Fig. 1. (PPTX 468 kb) Additional file 8: Table S3. Nucleotide substitution model selection implemented in DATAMONKEY. (XLSX 11 kb) Additional file 9: Table S4. Positively selected sites in IR-IFITM genes detected using FUBAR method implemented in DATAMONKEY. This summary table reports the means of posterior distribution of synonymous (α) and non-synonymous (β) substitution rates over sites, as well as the mean posterior probability for ω (=β/α) > 1 at a site. Indications of chain convergence and sampling variability for predictions at a given site are provided by the potential scale reduction factor (PSRF; if close to 1, then the MCMC chains have sufficiently converged), and the effective sample size Neff. (XLSX 13 kb) Additional file 10: Table S5. Site-specific tests for positive selection on different IFITM sub-families calculated using Codeml (PAML). lnL, the log-likelihood difference between the two models; 2DL, twice the log-likelihood difference between the two models. The positively selected sites were identified with posterior probability >0.95 using Bayes empirical Bayes (BEB) approach. *, posterior probability >0.95. Codon positions are according to the sequences of duck IFITM peptides. (XLSX 13 kb) Additional file 11: Table S6. Genes significantly differentially expressed in the lung and ileum of chicken and duck after infection with H5N1 and H5N2 avian influenza. (XLSX 821 kb) Additional file 12: Table S7. Pathway Express analysis of the chicken response to H5N1 infection in the ileum at 1dpi and H5N2 infection in the lung at 3dpi. The entries in red have a corrected gamma p value of <0.05; entries in blue have a value < 0.25 which is deemed to still be significant when using this software. (XLSX 30 kb) Additional file 13: Figure S6. Pathway Express analysis of the chicken response to HPAI infection in the ileum at 1dpi and to LPAI infection in the lung at 3dpi. In the ileum during H5N1 infection, genes involved in axon guidance are affected (A). In the lung during H5N2 infection, many genes involved in B- and T-cell receptor signalling are seen to be up-regulated (indicated in red). (B). and (C). (PPTX 156 kb) Additional file 14: Figure S7. Expander analysis of the chicken response to HPAI infection in the ileum at 1dpi and LPAI infection in the lung at day 3. Panels (A) and (B) refer to infection in the ileum and panels (C), (D) and (E) refer to infection in the lung (A). GO-terms associated with the genes which are being up-regulated (B). GO-terms associated with the genes which are being down-regulated. (C). GO-terms associated with the genes which are being up-regulated (D). GO-terms associated with the genes which are being down-regulated. Panel (E) shows an enrichment (*p* < 0.0001) of particular transcription factor binding sites amongst up-regulated genes. The frequency ratio (frequency in set divided by frequency in background) is shown. (PPTX 420 kb) Additional file 15: Figure S8. Pathway Express analysis of the duck response to HPAI infection in the ileum at 1dpi and in the lung at 3dpi. In the ileum, the TLR pathway is activated (A). By day 3 in the lung, several biological processes are being activated/inhibited. For example, extracellular matrix receptor interactions (B), and genes involved in leukocyte transendothelial migration (C). Many genes typically involved in cancer-associated pathways are also seen to be perturbed (D). Red indicates up-regulation and blue down-regulation. (PPTX 292 kb) Additional file 16: Table S8. Pathway Express analysis of the duck response to H5N1 infection in the ileum at 1dpi (A) and in the lung at 3dpi (B). (XLSX 27 kb) Additional file 17: Figure S9. Expander analysis of the duck response to HPAI infection in the ileum at 1dpi and in the lung at 3dpi. Panels (A) and (B) refer to infection in the ileum and panels (C), (D), (E) and (F) refer to infection in the lung. (A). GO-terms associated with the genes which are being up-regulated. Panel (B) shows an enrichment (*p* < 0.0001) of IRF7 transcription factor binding sites amongst up-regulated genes. The frequency ratio (frequency in set divided by frequency in background) is shown. (C). GO-terms associated with the genes which are being up-regulated (D). GO-terms associated with the genes which are being down-regulated. Panel (E) shows an enrichment (*p* < 0.0001) of genes residing on chromosome 1 amongst the up-regulated genes. Panel (F) shows an enrichment (*p* < 0.0001) of NF-kB transcription factor binding sites amongst up-regulated genes. The frequency ratio (frequency in set divided by frequency in background) is shown. (PPTX 369 kb) Additional file 18: Figure S10. Ingenuity Pathway Analysis (IPA) analysis of host-specific responses to influenza infection in the lung at day 3 pi. (A). The unique chicken response to H5N2 is overwhelmingly concerned with T- and B-cell development and activation and cell death (B). Biological network showing genes involved in the cell-mediated immune response which is seen in the chicken after H5N2 infection. (C). The duck response to H5N1 infection is seen to be concerned with pathogen-associated molecular patterns and the RIG-I and TLR pathways. (PPTX 292 kb) Additional file 19: Figure S11. IFITM gene expression in lung tissue measured by qRT-PCR in three control and three HPAI H5N1 (A/Vietnam/1203/04) infected chicken and duck samples 1 dpi. IFITM gene expression was measured by Taqman® qRT-PCR and data were normalised to the endogenous control gene eukaryotic 18S rRNA to generate ΔCt values. Data are presented as 30-ΔCt so that an increase in 30-ΔCt represents an increase in gene expression. (PPTX 557 kb) Additional file 20: Table S9. DAVID analysis of genes clustering with expression of IFITM1 and IFITM2. (XLSX 16 kb) Additional file 21: Table S10. DAVID analysis of genes clustering with expression of IFITM3. (XLSX 17 kb) Additional file 22: Table S11. Gene-specific primers used in cloning the duck IFITM gene sequences. (XLSX 12 kb)

## Abbreviations

AI: Avian influenza
BEB: Bayes Empirical Bayes
Chr: Chromosome
CID_50_: 50 % Chicken infectious dose
CIL: Conserved intracellular loop
DE: Differentially expressed
dpi: Days post infection
EID_50_: 50 % Egg infectious dose
FC: Fold change
FDR: False discovery rate
GO: Gene ontology
HP: Highly pathogenic
IR-IFITM: Immune related interferon induced transmembrane proteins
LP: Low pathogenic
Mya: Million years ago
PCR: Polymerase chain reaction
PRR: Pattern recognition receptor
QRT-PCR: Quantitative reverse transcription polymerase chain reaction
RT: Reverse transcription
ssRNA: single stranded RNA
TFBS: Transcription factor binding site
TM: Transmembrane
