# Supplementary material for: A comparative analysis of host responses to avian influenza infection in ducks and chickens highlights a role for the interferon-induced transmembrane proteins in viral resistance
Source: BMC Genomics. 2015 Aug 4;16(1):574. doi: 10.1186/s12864-015-1778-8 (PMC4523026; doi:10.1186/s12864-015-1778-8)
Supplement: Additional file 1: Figure S1. — Domain structure of the chicken and duck IFITM proteins as determined by the SMART algorithm [47]. Blue blocks show the transmembrane regions while pink defines areas of low complexity. The resultant coding sequences were as follows: IFITM1 - 420 nucleotides (140 amino acids); IFITM2 - 327 nucleotides (109 amino acids); IFITM3 - 429 nucleotides (143 amino acids); IFITM5 - 402 nucleotides (134 amino acids); IFITM10 - 591 nucleotides (197 amino acids). (PPTX 84 kb) [file 12864_2015_1778_MOESM1_ESM.pptx]

## Slide 1
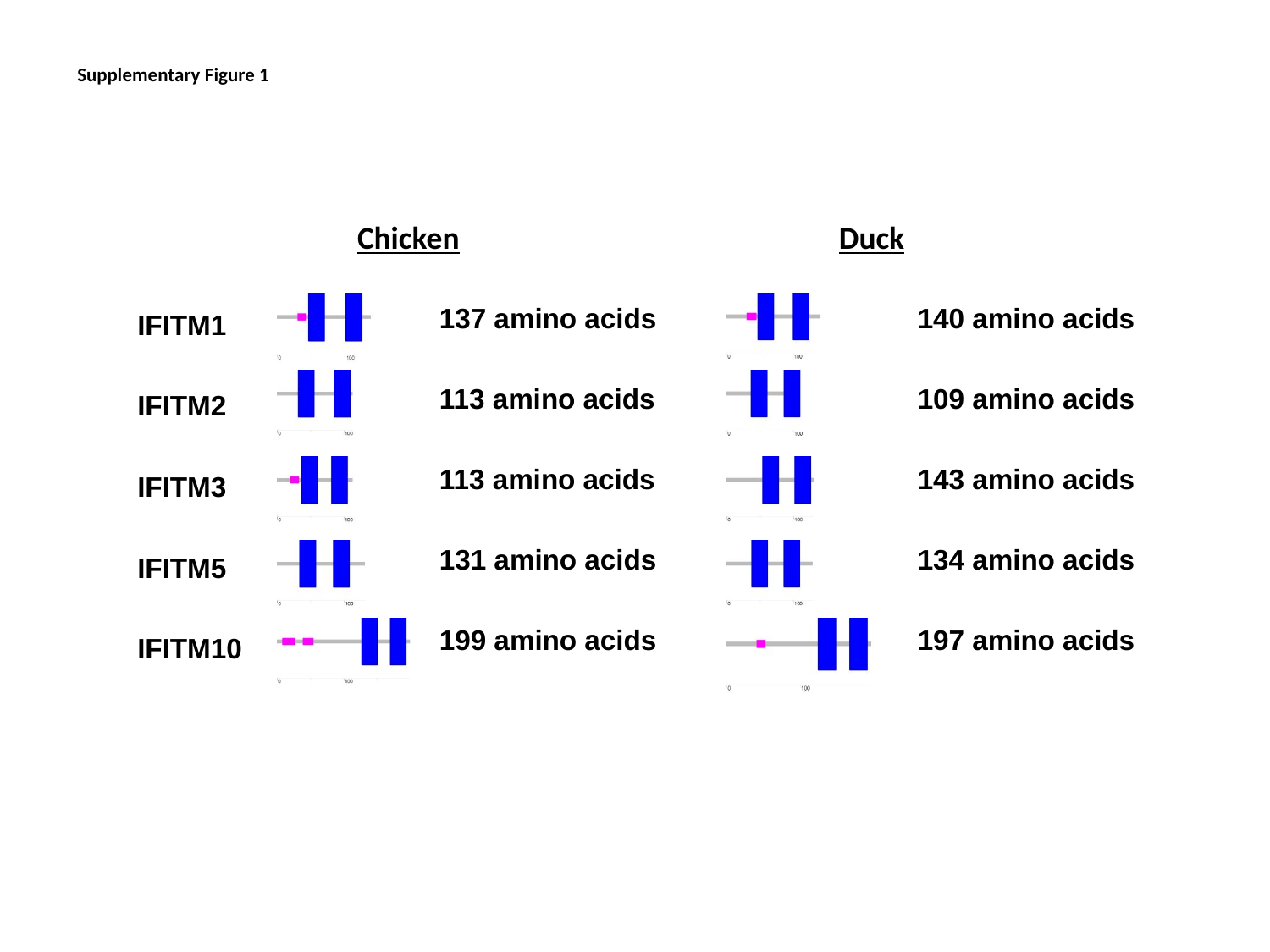

Supplementary Figure 1
Chicken
Duck
137 amino acids
140 amino acids
IFITM1
113 amino acids
109 amino acids
IFITM2
113 amino acids
143 amino acids
IFITM3
131 amino acids
134 amino acids
IFITM5
199 amino acids
197 amino acids
IFITM10
