# Supplementary material for: A comparative analysis of host responses to avian influenza infection in ducks and chickens highlights a role for the interferon-induced transmembrane proteins in viral resistance
Source: BMC Genomics. 2015 Aug 4;16(1):574. doi: 10.1186/s12864-015-1778-8 (PMC4523026; doi:10.1186/s12864-015-1778-8)
Supplement: Additional file 5: Figure S3. — Molecular Phylogenetic analysis by Maximum Likelihood method of IFIT proteins using MSA in Additional file 4: Figure S2. The evolutionary history was inferred by using the Maximum Likelihood method based on the JTT matrix-based model. The bootstrap consensus tree inferred from 1000 replicates is taken to represent the evolutionary history of the taxa analysed. Branches corresponding to partitions reproduced in less than 50 % bootstrap replicates are collapsed. The percentage of replicate trees in which the associated taxa clustered together in the bootstrap test (1000 replicates) are shown next to the branches. Initial tree(s) for the heuristic search were obtained automatically by applying Neighbor-Join and BioNJ algorithms to a matrix of pairwise distances estimated using a JTT model, and then selecting the topology with superior log likelihood value. A discrete Gamma distribution was used to model evolutionary rate differences among sites (5 categories (+G, parameter = 3.0096)). The analysis involved 205 amino acid sequences. All positions with less than 95 % site coverage were eliminated. That is, fewer than 5 % alignment gaps, missing data, and ambiguous bases were allowed at any position. There were a total of 98 positions in the final dataset. Evolutionary analyses were conducted in MEGA6. (PPTX 226 kb) [file 12864_2015_1778_MOESM5_ESM.pptx]

## Slide 1
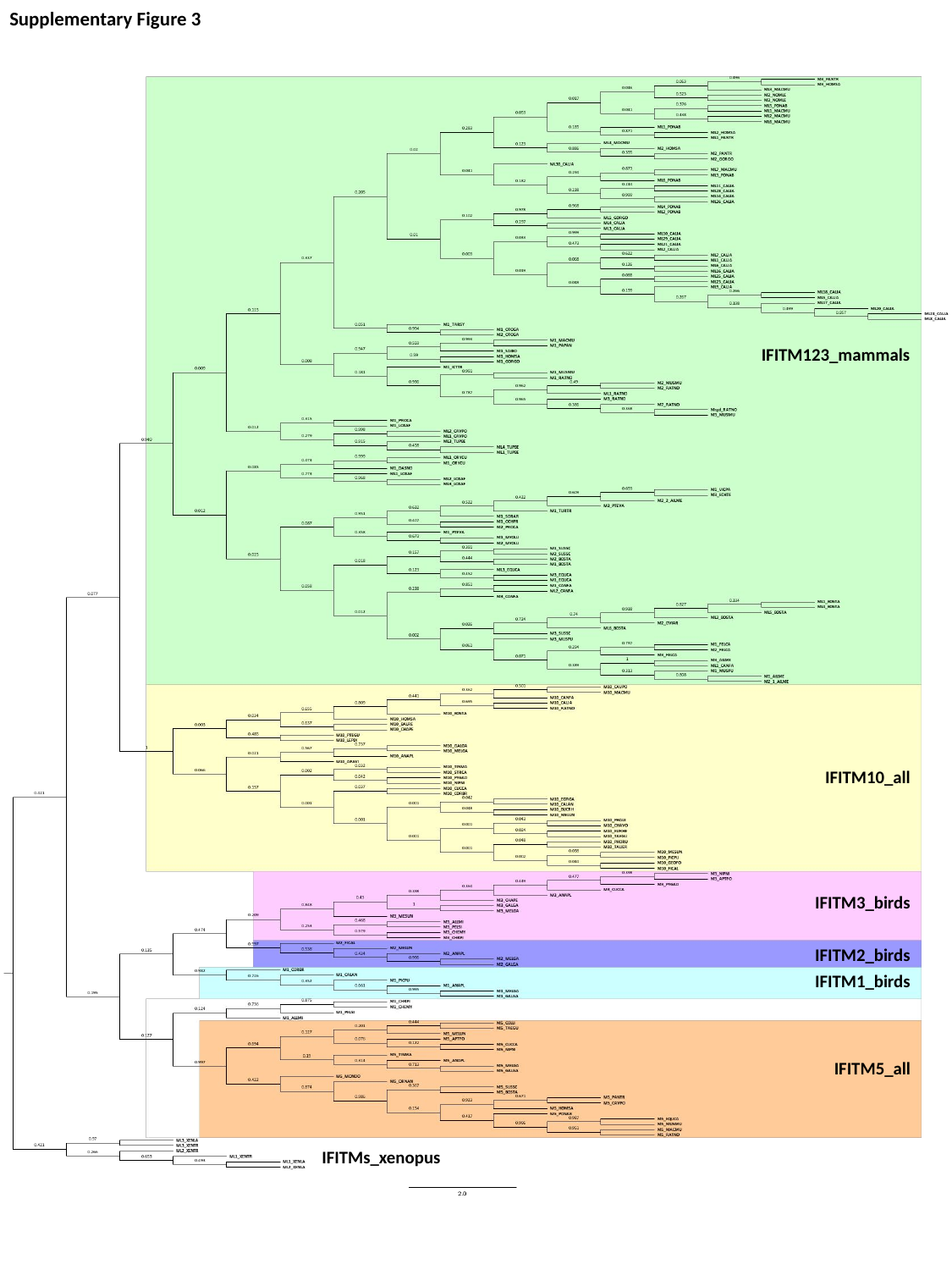

Supplementary Figure 3
IFITM123_mammals
IFITM10_all
IFITM3_birds
IFITM2_birds
IFITM1_birds
IFITM5_all
IFITMs_xenopus
