# Supplementary figures and images for: A comparative analysis of host responses to avian influenza infection in ducks and chickens highlights a role for the interferon-induced transmembrane proteins in viral resistance
Source: BMC Genomics. 2015 Aug 4;16(1):574. doi: 10.1186/s12864-015-1778-8 (PMC4523026; doi:10.1186/s12864-015-1778-8)

## Slide 1
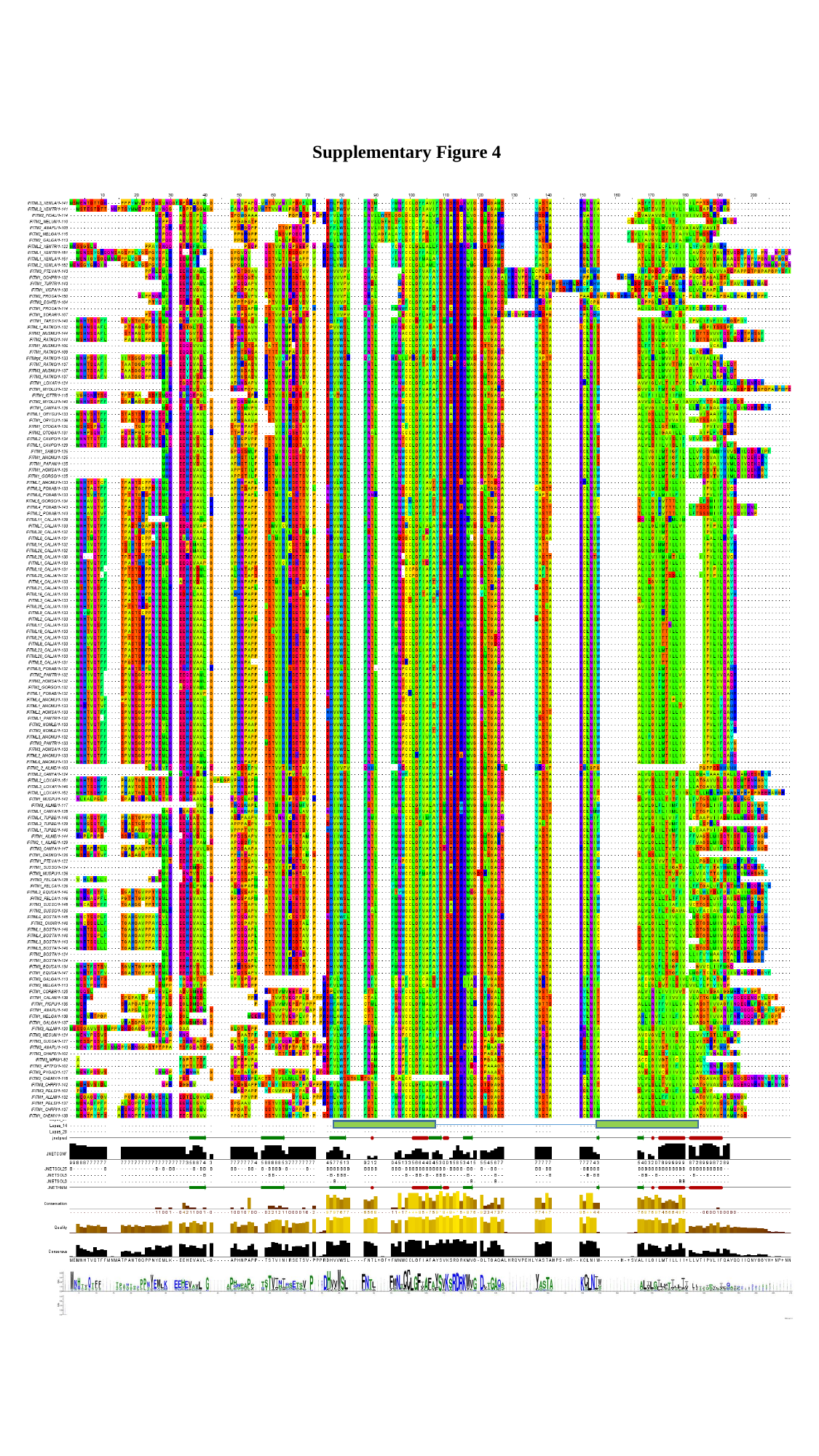

Supplementary Figure 4

Supplement: Additional file 6: Figure S4. — Multiple sequence alignment of IFITM1, 2 and 3-like proteins using MUSCLE. The transmembrane domains and other features are shown at the foot of the figure, and were displayed using options from Jalview. For more details see legend to Fig. 6. (PPTX 801 kb) [file 12864_2015_1778_MOESM6_ESM.pptx]

## Slide 1
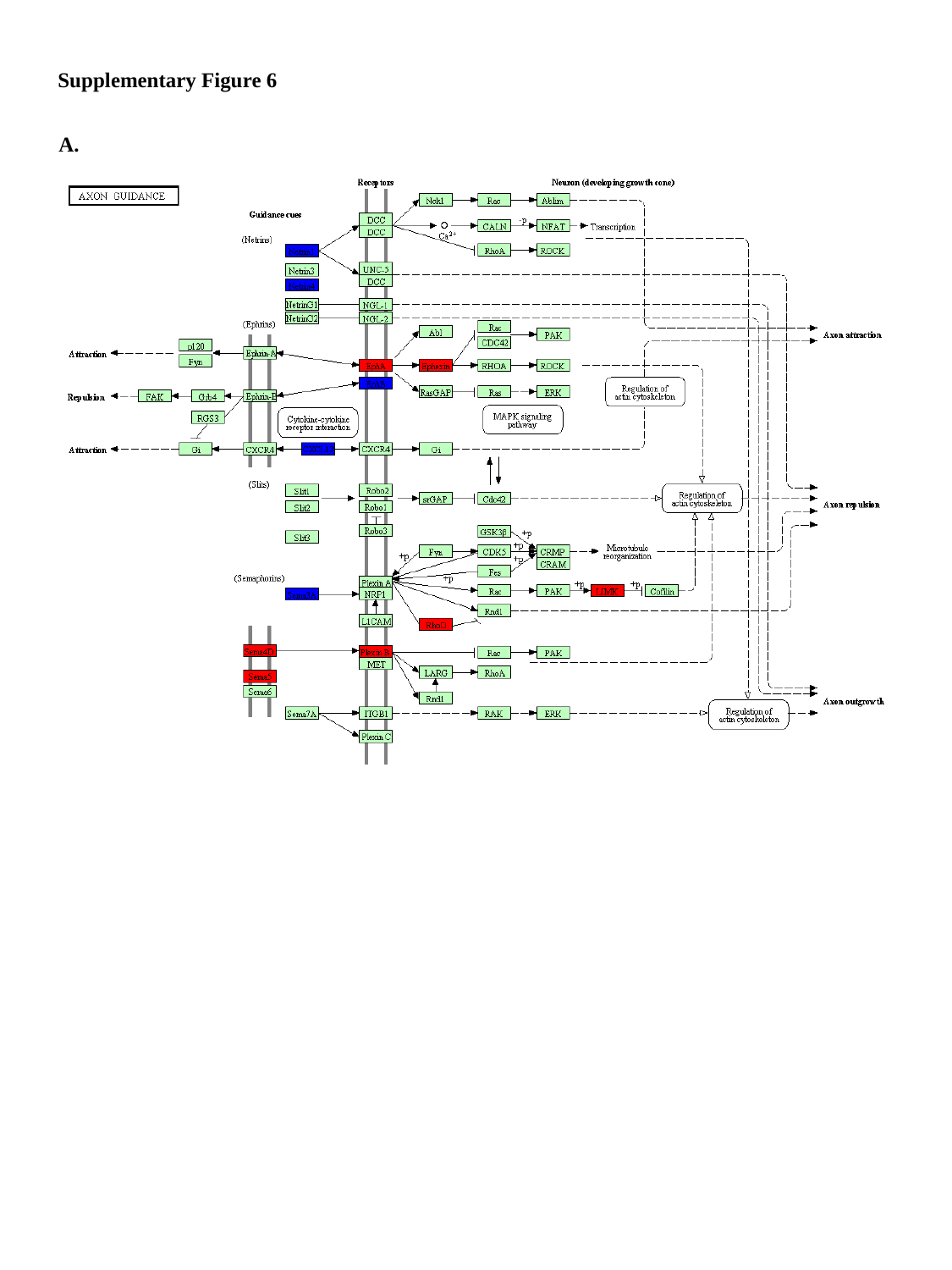

Supplementary Figure 6
A.

## Slide 2
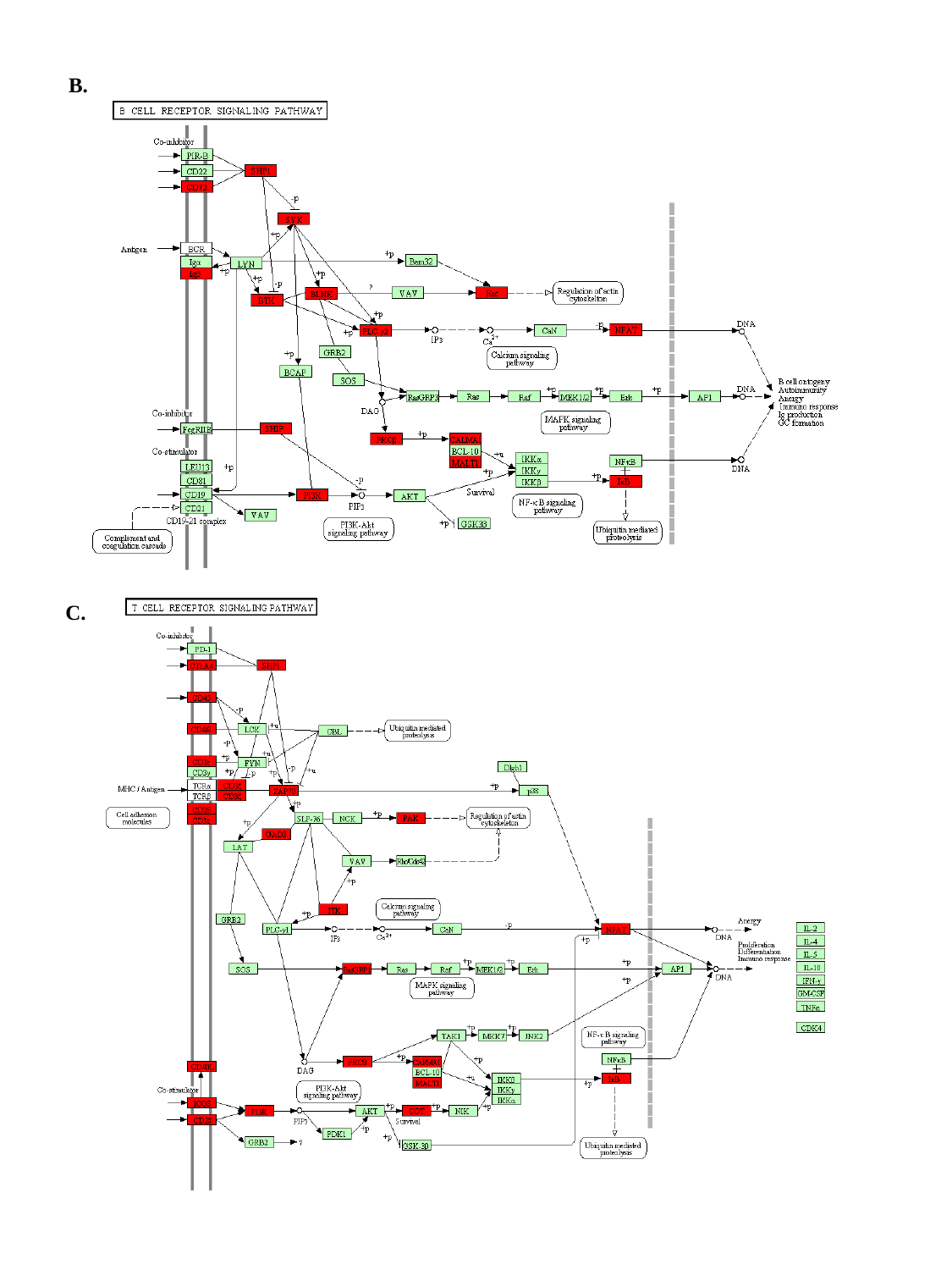

B.
C.

Supplement: Additional file 13: Figure S6. — Pathway Express analysis of the chicken response to HPAI infection in the ileum at 1dpi and to LPAI infection in the lung at 3dpi. In the ileum during H5N1 infection, genes involved in axon guidance are affected (A). In the lung during H5N2 infection, many genes involved in B- and T-cell receptor signalling are seen to be up-regulated (indicated in red). (B). and (C). (PPTX 156 kb) [file 12864_2015_1778_MOESM13_ESM.pptx]

## Slide 1
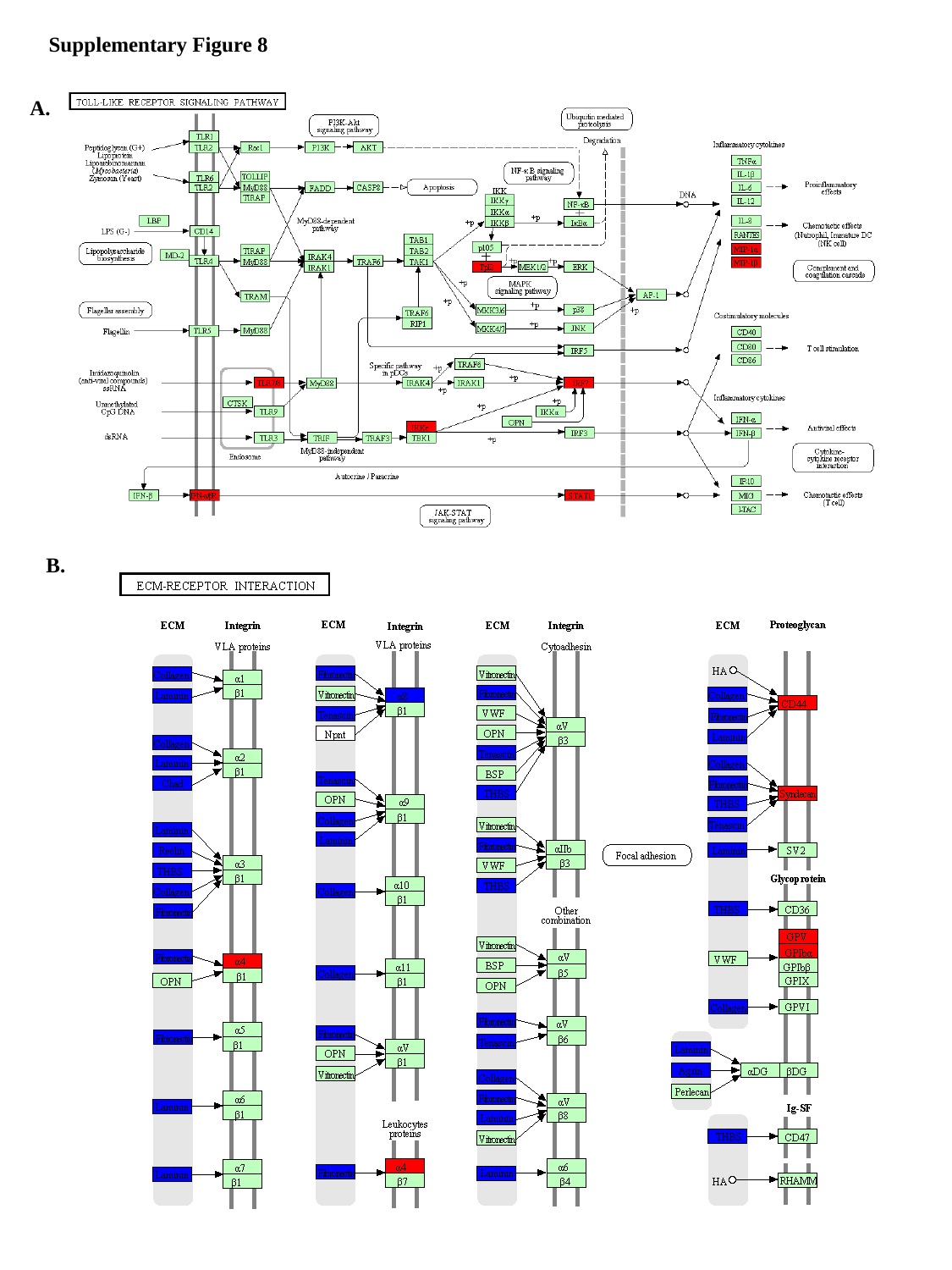

Supplementary Figure 8
A.
B.

## Slide 2
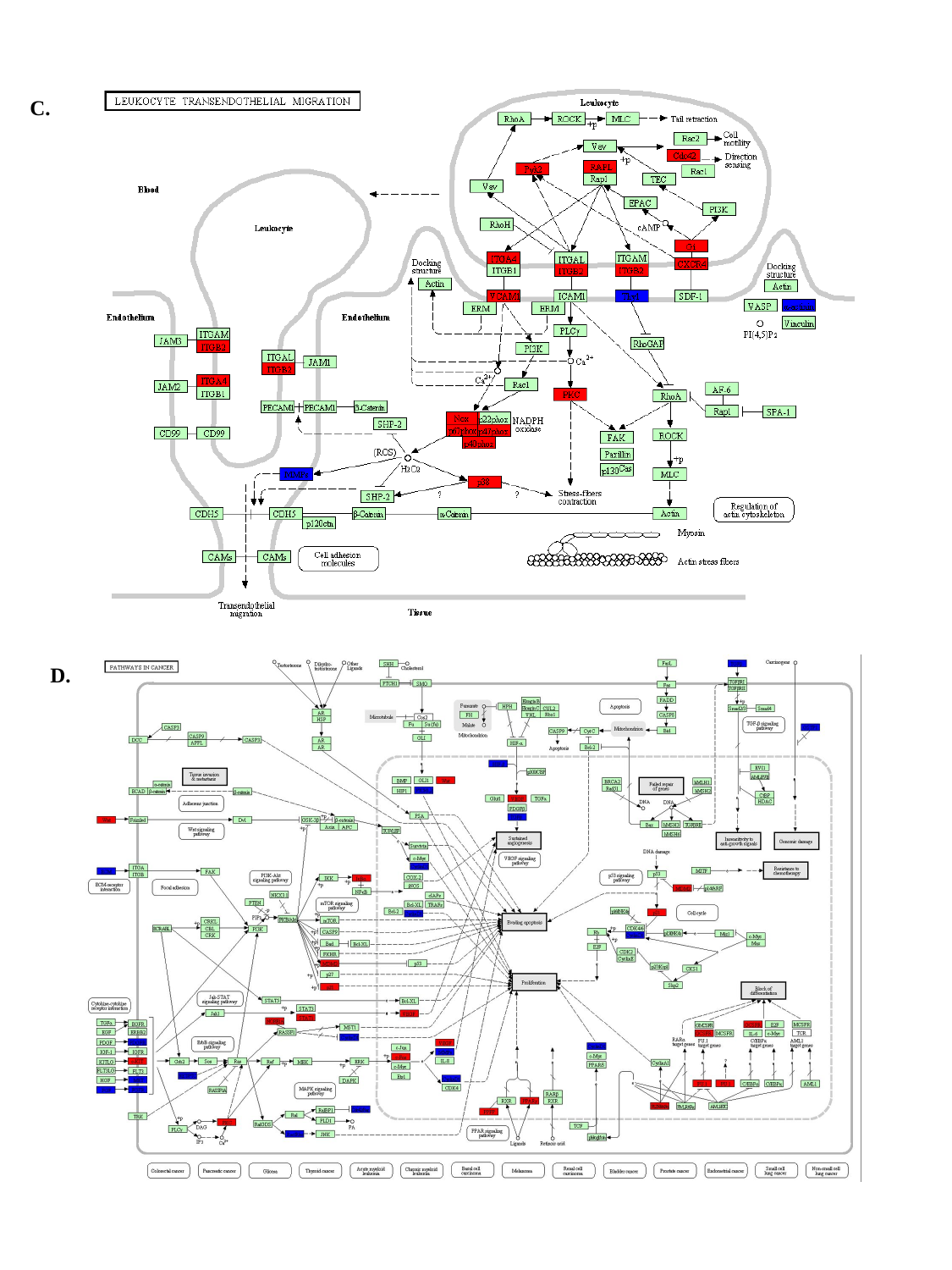

C.
D.

Supplement: Additional file 15: Figure S8. — Pathway Express analysis of the duck response to HPAI infection in the ileum at 1dpi and in the lung at 3dpi. In the ileum, the TLR pathway is activated (A). By day 3 in the lung, several biological processes are being activated/inhibited. For example, extracellular matrix receptor interactions (B), and genes involved in leukocyte transendothelial migration (C). Many genes typically involved in cancer-associated pathways are also seen to be perturbed (D). Red indicates up-regulation and blue down-regulation. (PPTX 292 kb) [file 12864_2015_1778_MOESM15_ESM.pptx]

## Slide 1
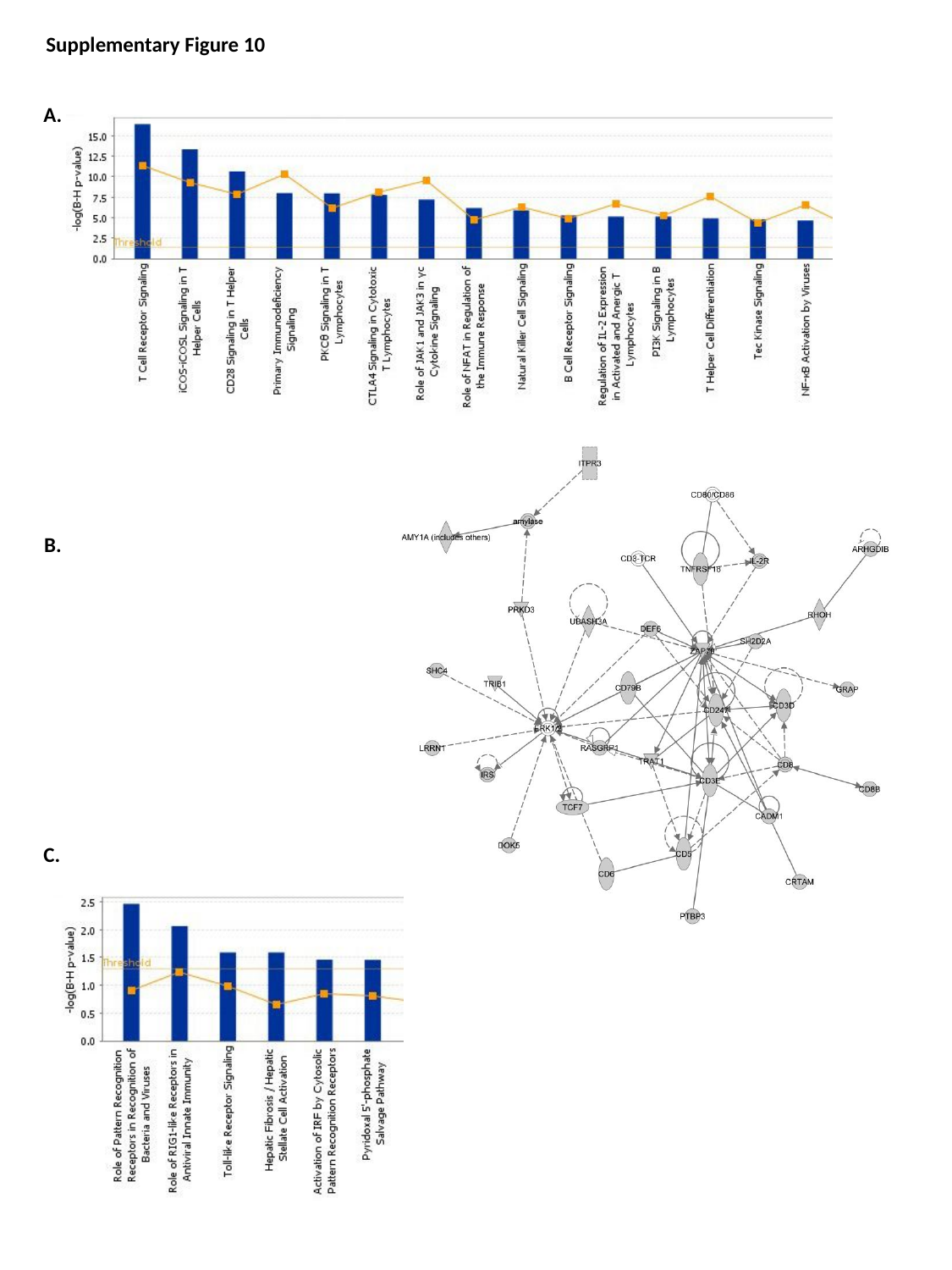

Supplementary Figure 10
A.
B.
C.

Supplement: Additional file 18: Figure S10. — Ingenuity Pathway Analysis (IPA) analysis of host-specific responses to influenza infection in the lung at day 3 pi. (A). The unique chicken response to H5N2 is overwhelmingly concerned with T- and B-cell development and activation and cell death (B). Biological network showing genes involved in the cell-mediated immune response which is seen in the chicken after H5N2 infection. (C). The duck response to H5N1 infection is seen to be concerned with pathogen-associated molecular patterns and the RIG-I and TLR pathways. (PPTX 292 kb) [file 12864_2015_1778_MOESM18_ESM.pptx]

## Slide 1
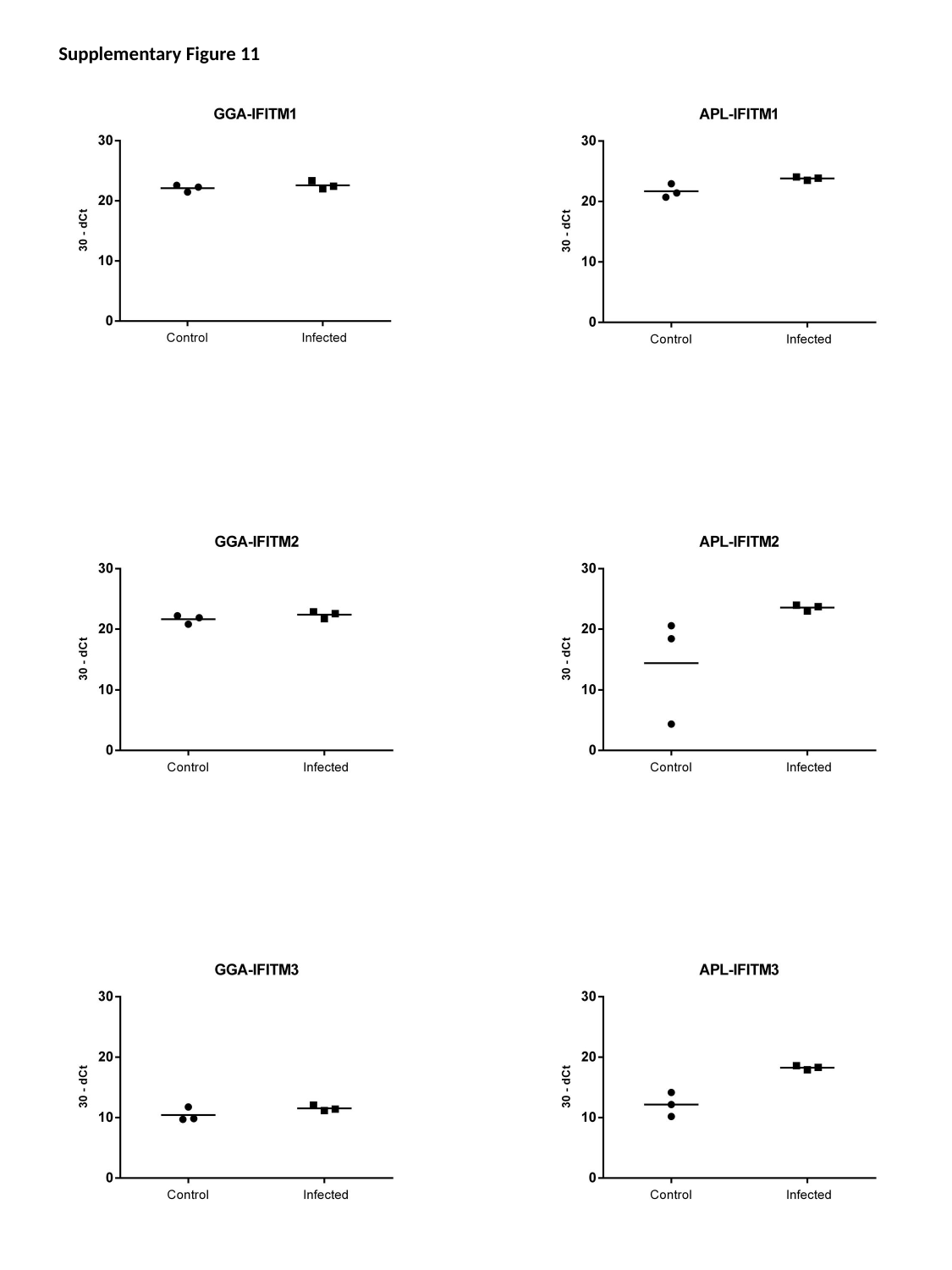

Supplementary Figure 11

Supplement: Additional file 19: Figure S11. — IFITM gene expression in lung tissue measured by qRT-PCR in three control and three HPAI H5N1 (A/Vietnam/1203/04) infected chicken and duck samples 1 dpi. IFITM gene expression was measured by Taqman® qRT-PCR and data were normalised to the endogenous control gene eukaryotic 18S rRNA to generate ΔCt values. Data are presented as 30-ΔCt so that an increase in 30-ΔCt represents an increase in gene expression. (PPTX 557 kb) [file 12864_2015_1778_MOESM19_ESM.pptx]
