# Supplementary material for: A comparative analysis of host responses to avian influenza infection in ducks and chickens highlights a role for the interferon-induced transmembrane proteins in viral resistance
Source: BMC Genomics. 2015 Aug 4;16(1):574. doi: 10.1186/s12864-015-1778-8 (PMC4523026; doi:10.1186/s12864-015-1778-8)
Supplement: Additional file 7: Figure S5. — Evolutionary relationships of IFITM1, 2 and 3 proteins in vertebrates. (A) The evolutionary history was inferred using the Maximum Parsimony method (PAUP). The bootstrap consensus tree inferred from 500 replicates is taken to represent the evolutionary history of the taxa analysed. Branches corresponding to partitions reproduced in less than 50 % bootstrap replicates are collapsed. The percentage of replicate trees in which the associated taxa clustered together in the bootstrap test (500 replicates) are shown next to the branches. The MP tree was obtained using the Subtree-Pruning-Regrafting (SPR) algorithm with search level 1 in which the initial trees were obtained by the random addition of sequences (10 replicates). The analysis involved 148 amino acid sequences. All positions with less than 95 % site coverage were eliminated. That is, fewer than 5 % alignment gaps, missing data, and ambiguous bases were allowed at any position. There were a total of 86 positions in the final dataset. Evolutionary analyses were conducted in MEGA6. (B) The evolutionary history was inferred using the Neighbour-Joining method (NJ). The bootstrap consensus tree inferred from 500 replicates is taken to represent the evolutionary history of the taxa analysed. Branches corresponding to partitions reproduced in less than 50 % bootstrap replicates are collapsed. The percentage of replicate trees in which the associated taxa clustered together in the bootstrap test (500 replicates) are shown next to the branches. The evolutionary distances were computed using the JTT matrix-based method and are in the units of the number of amino acid substitutions per site. The rate variation among sites was modelled with a gamma distribution (shape parameter = 5). The analysis involved 148 amino acid sequences. All positions with less than 95 % site coverage were eliminated. That is, fewer than 5 % alignment gaps, missing data, and ambiguous bases were allowed at any position. There were a total of 86 p [file 12864_2015_1778_MOESM7_ESM.pptx]

## Slide 1
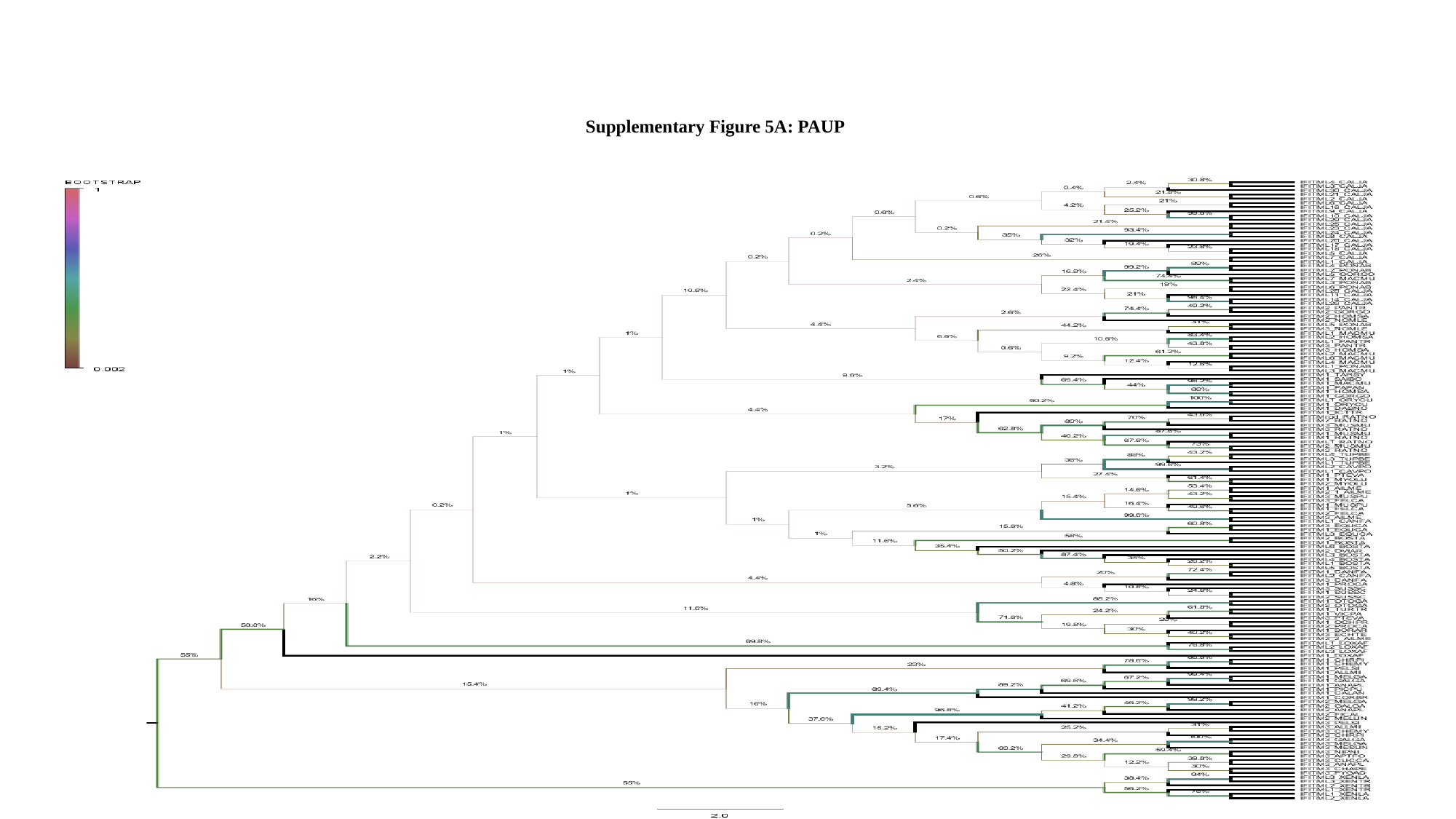

Supplementary Figure 5A: PAUP

## Slide 2
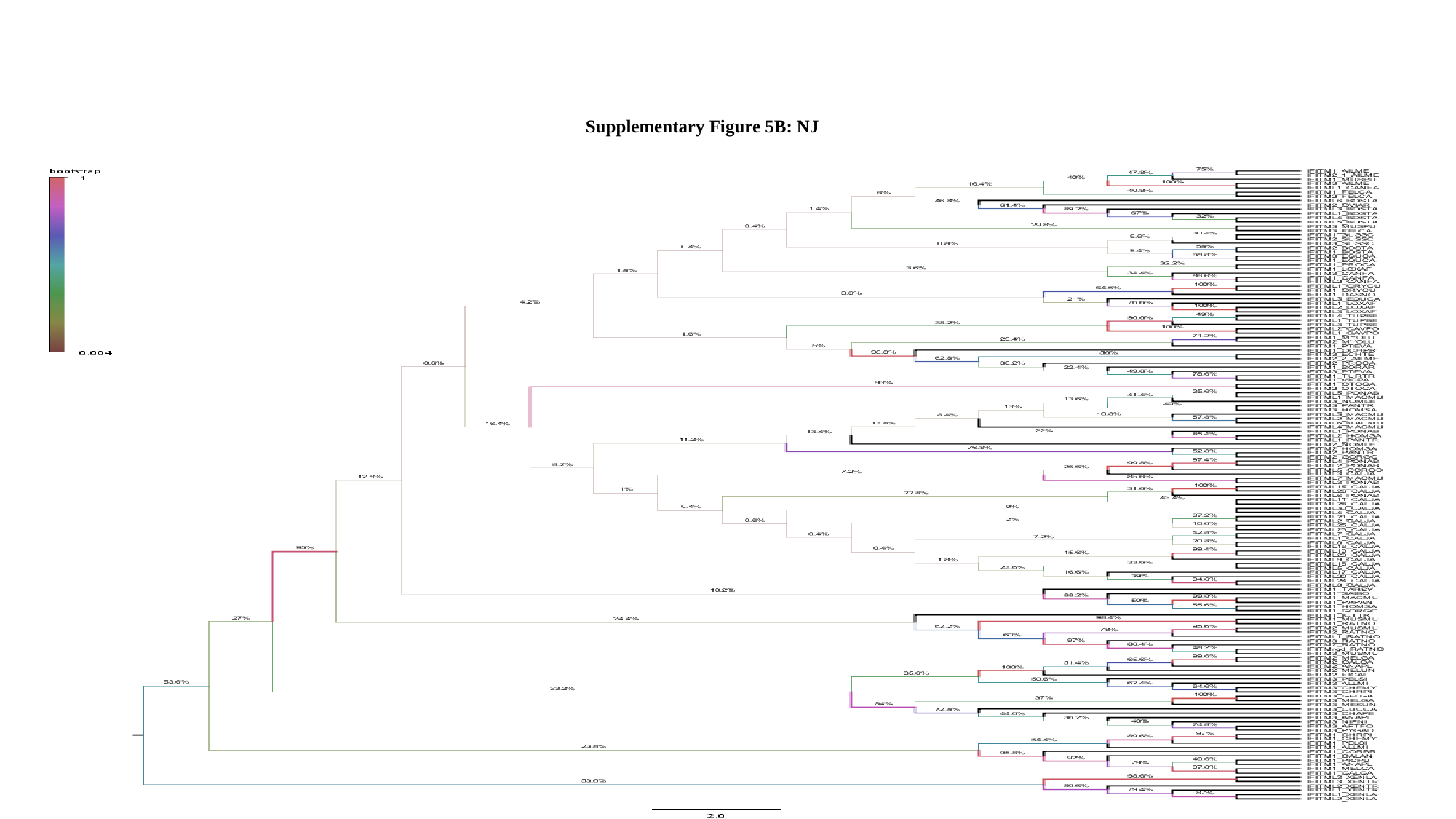

Supplementary Figure 5B: NJ

## Slide 3
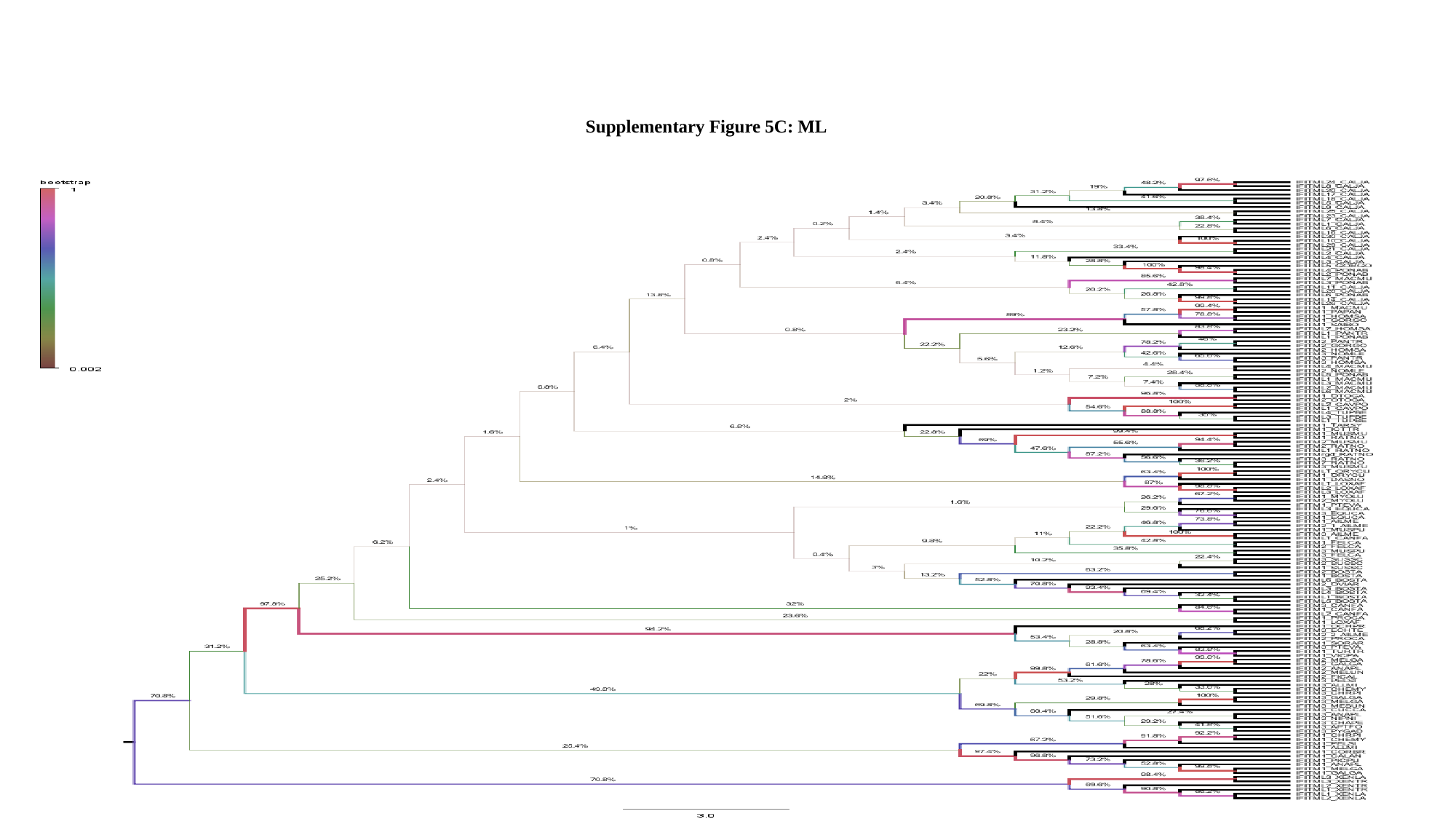

Supplementary Figure 5C: ML

## Slide 4
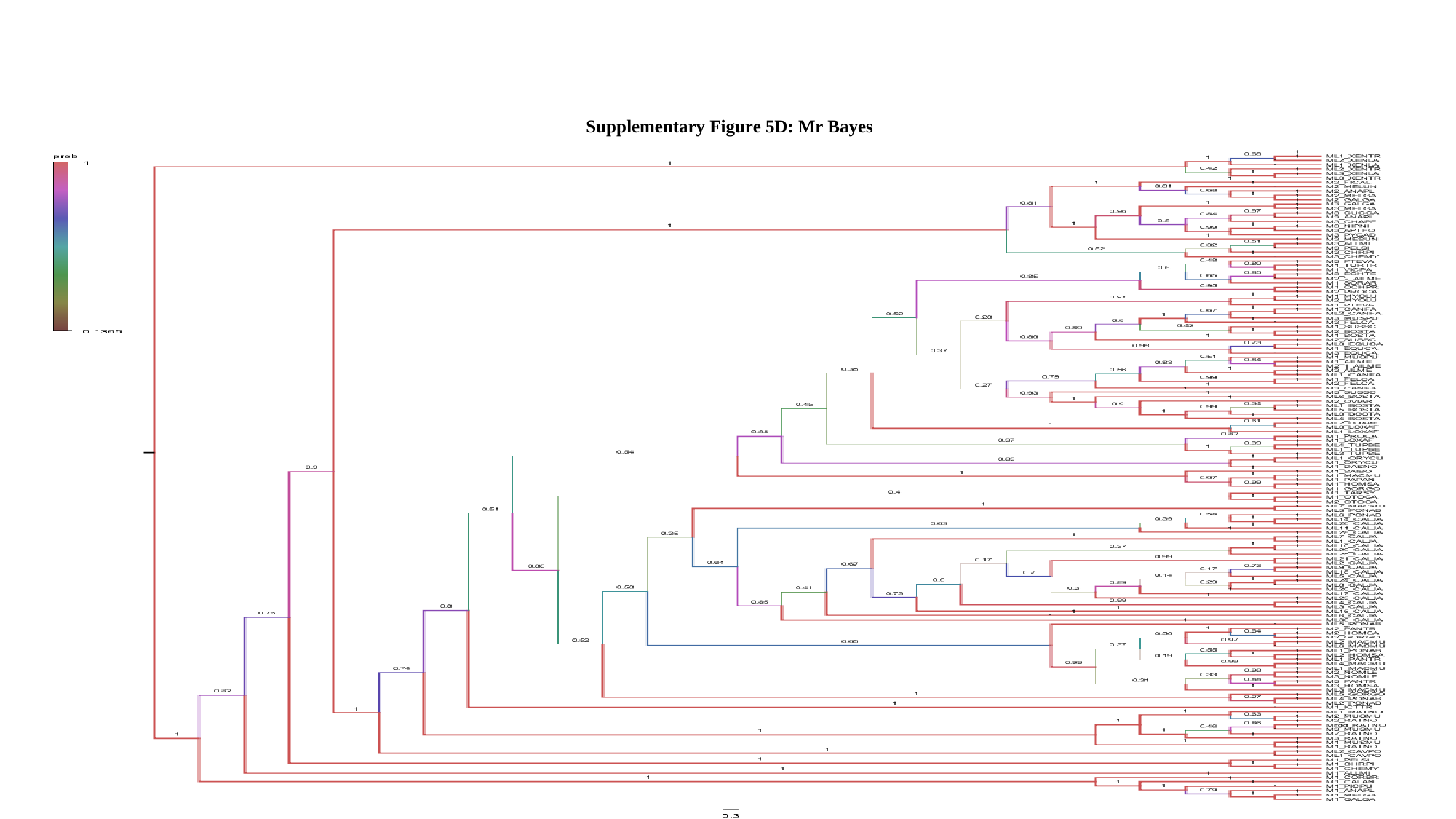

Supplementary Figure 5D: Mr Bayes
