# Supplementary material for: A comparative analysis of host responses to avian influenza infection in ducks and chickens highlights a role for the interferon-induced transmembrane proteins in viral resistance
Source: BMC Genomics. 2015 Aug 4;16(1):574. doi: 10.1186/s12864-015-1778-8 (PMC4523026; doi:10.1186/s12864-015-1778-8)
Supplement: Additional file 14: Figure S7. — Expander analysis of the chicken response to HPAI infection in the ileum at 1dpi and LPAI infection in the lung at day 3. Panels (A) and (B) refer to infection in the ileum and panels (C), (D) and (E) refer to infection in the lung (A). GO-terms associated with the genes which are being up-regulated (B). GO-terms associated with the genes which are being down-regulated. (C). GO-terms associated with the genes which are being up-regulated (D). GO-terms associated with the genes which are being down-regulated. Panel (E) shows an enrichment (p < 0.0001) of particular transcription factor binding sites amongst up-regulated genes. The frequency ratio (frequency in set divided by frequency in background) is shown. (PPTX 420 kb) [file 12864_2015_1778_MOESM14_ESM.pptx]

## Slide 1
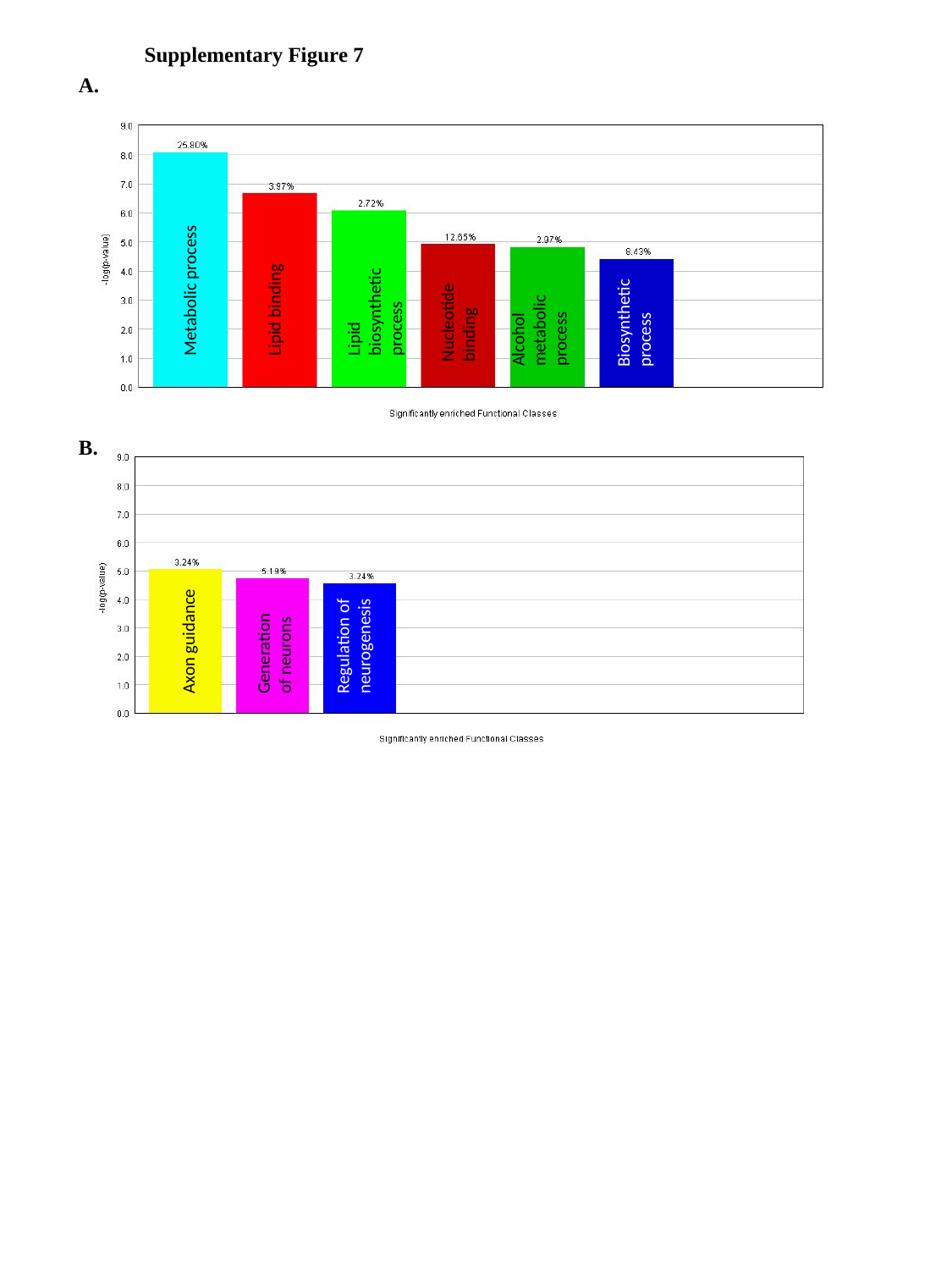

Supplementary Figure 7
A.
Metabolic process
Lipid binding
Lipid biosynthetic process
Nucleotide binding
Alcohol metabolic process
Biosynthetic process
ELF1
B.
Axon guidance
Regulation of neurogenesis
Generation of neurons
Chromosome Z

## Slide 2
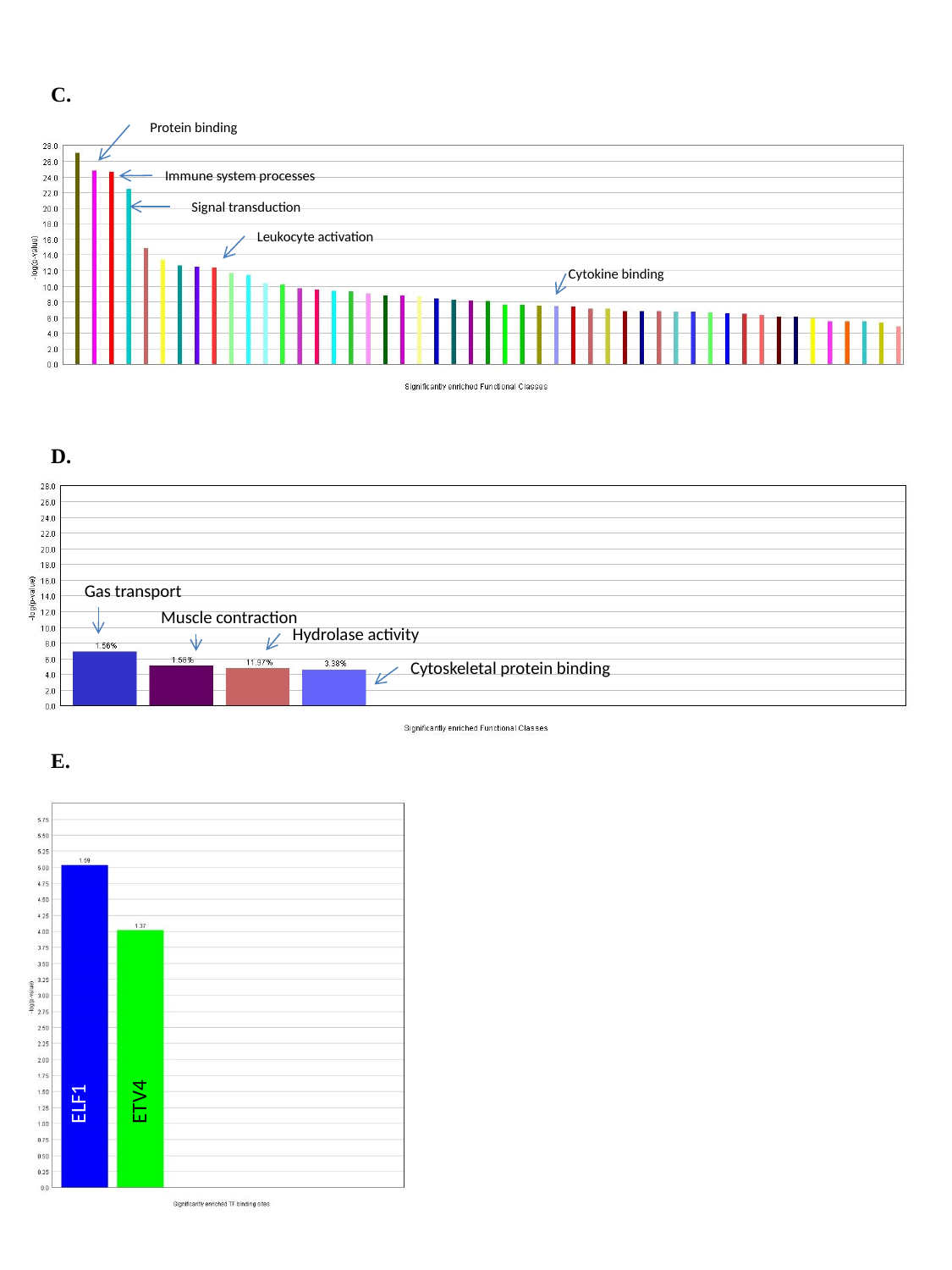

C.
Protein binding
Immune system processes
Signal transduction
Leukocyte activation
Cytokine binding
D.
Gas transport
Muscle contraction
Hydrolase activity
Cytoskeletal protein binding
E.
ELF1
ETV4
