# Supplementary material for: A comparative analysis of host responses to avian influenza infection in ducks and chickens highlights a role for the interferon-induced transmembrane proteins in viral resistance
Source: BMC Genomics. 2015 Aug 4;16(1):574. doi: 10.1186/s12864-015-1778-8 (PMC4523026; doi:10.1186/s12864-015-1778-8)
Supplement: Additional file 17: Figure S9. — Expander analysis of the duck response to HPAI infection in the ileum at 1dpi and in the lung at 3dpi. Panels (A) and (B) refer to infection in the ileum and panels (C), (D), (E) and (F) refer to infection in the lung. (A). GO-terms associated with the genes which are being up-regulated. Panel (B) shows an enrichment (p < 0.0001) of IRF7 transcription factor binding sites amongst up-regulated genes. The frequency ratio (frequency in set divided by frequency in background) is shown. (C). GO-terms associated with the genes which are being up-regulated (D). GO-terms associated with the genes which are being down-regulated. Panel (E) shows an enrichment (p < 0.0001) of genes residing on chromosome 1 amongst the up-regulated genes. Panel (F) shows an enrichment (p < 0.0001) of NF-kB transcription factor binding sites amongst up-regulated genes. The frequency ratio (frequency in set divided by frequency in background) is shown. (PPTX 369 kb) [file 12864_2015_1778_MOESM17_ESM.pptx]

## Slide 1
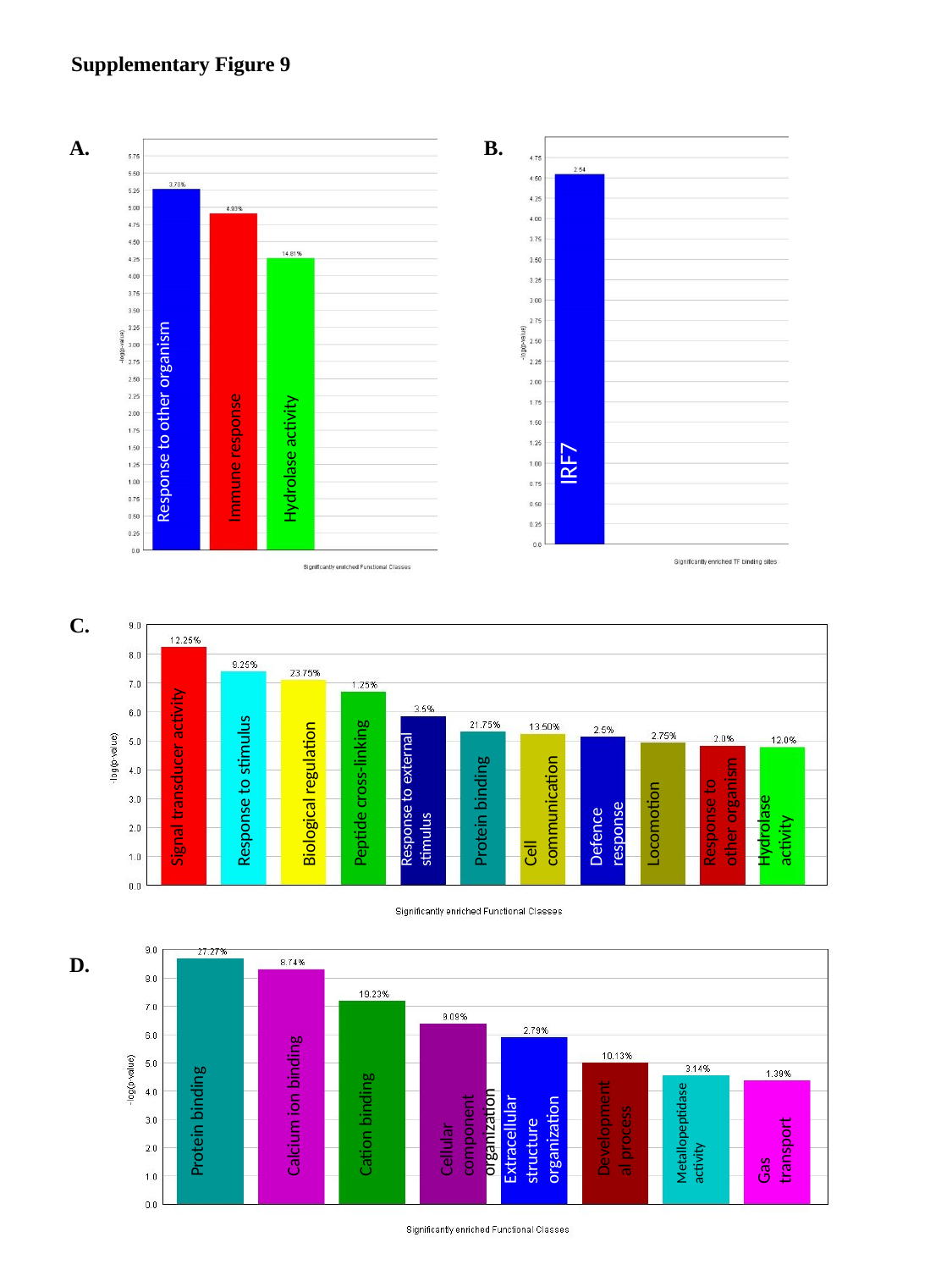

Supplementary Figure 9
A.
B.
Response to other organism
Immune response
Hydrolase activity
IRF7
C.
Signal transducer activity
Response to stimulus
Biological regulation
Peptide cross-linking
Response to external stimulus
Protein binding
Cell communication
Defence response
Locomotion
Response to other organism
Hydrolase activity
D.
Protein binding
Calcium ion binding
Cation binding
Cellular component organization
Extracellular structure organization
Metallopeptidase activity
Developmental process
Gas transport

## Slide 2
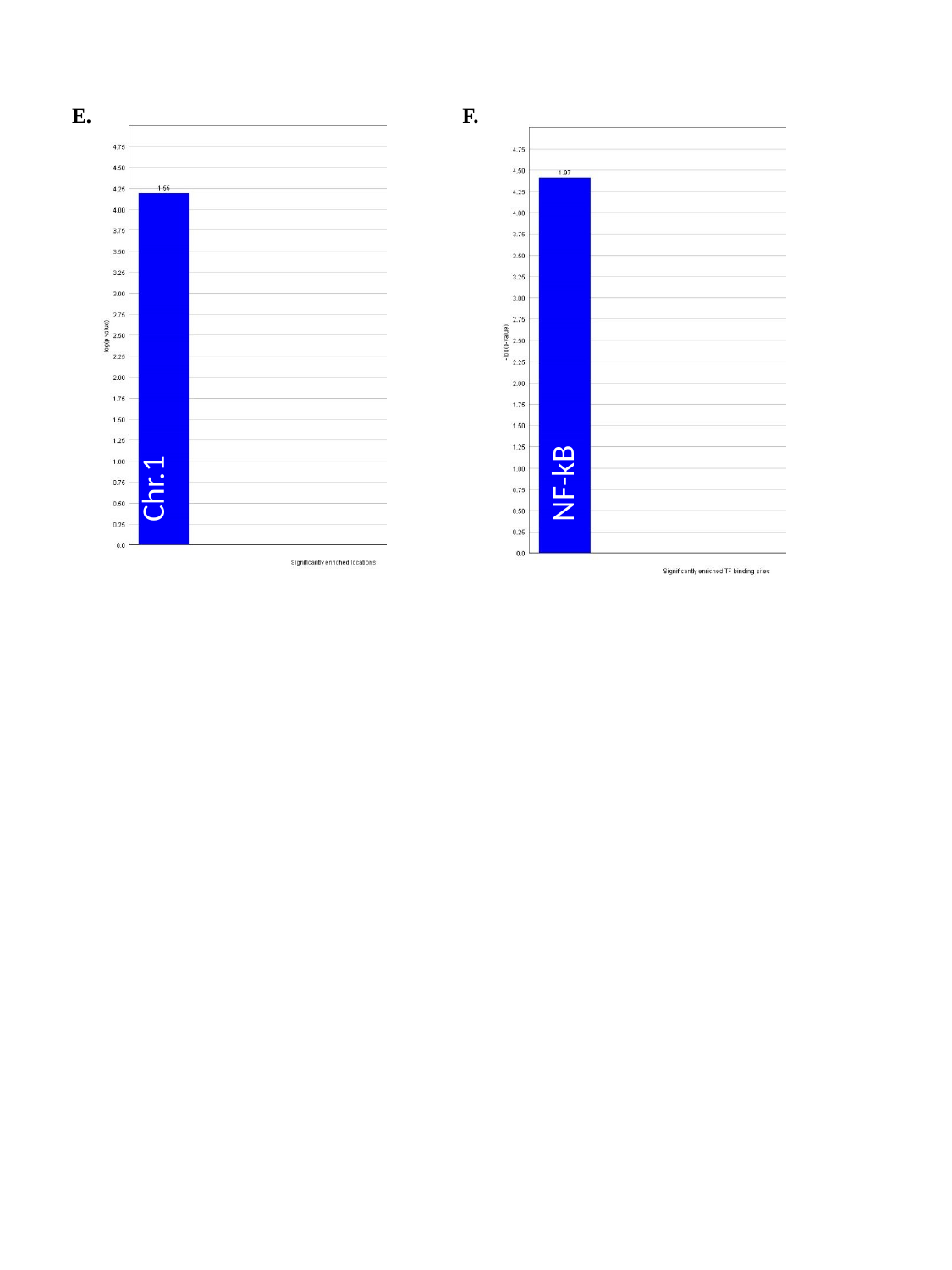

E.
F.
NF-kB
Chr.1
